# Deciphering defensive mechanisms in *Riemerella anatipestifer* infection: pathogen adaptation, host immune response and emerging strategies

**DOI:** 10.3389/fimmu.2026.1929344

**Published:** 2026-09-08

**Authors:** Rochelle A. Flores, Cherry P. Fernandez-Colorado, Paula Leona C. Fletcher, Andrea Gail M. Villavicencio, Woo H. Kim, Wongi Min

**Affiliations:** 1 College of Veterinary Medicine & Institute of Animal Medicine, Gyeongsang National University, Jinju, Republic of Korea; 2 Department of Veterinary Paraclinical Sciences, College of Veterinary Medicine, University of the Philippines Los Baños, College, Los Baños, Laguna, Philippines; 3 Virology and Vaccine Institute of the Philippines Program, Industrial Technology Development Institute, Department of Science and Technology, Bicutan, Taguig, Philippines; 4 Department of Medical Microbiology, College of Public Health, University of the Philippines Manila, Ermita, Manila, Philippines; 5 Hoxbio, Business Center, Gyeongsang National University, Jinju, Republic of Korea

**Keywords:** avian immunology, host-pathogen interaction, immunopathogenesis, multidrug resistance, protective immunity, *Riemerella anatipestifer*, vaccine development

## Abstract

*Riemerella anatipestifer* (RA) is an economically important avian pathogen that has historically caused significant disease in waterfowls but is increasingly recognized in various poultry production systems and wild bird species, highlighting its expanding host range and adaptive potential. Despite the availability of vaccines and antimicrobial interventions, RA remains a persistent challenge due to serotype diversity, limited cross-protective immunity, evolving antimicrobial resistance patterns, and an incomplete understanding of the host-pathogen interactions that govern disease outcome. Recent advances in genomics, molecular epidemiology, immunology and vaccinology have generated new insights into RA evolution, pathogenicity, host adaptation, and immune protection. However, these advances remain fragmented, hindering the translation of mechanistic discoveries into broadly effective control strategies. Emerging evidence further indicates that disease progression is shaped not only by bacterial pathogenicity but also by the magnitude and regulation of host immune responses, where dysregulated inflammatory signaling and immune-mediated tissue damage contribute significantly to pathology. This review synthesizes current understanding of the dynamic interplay between pathogen adaptation, immune evasion, host defense responses, and immunopathogenesis that collectively determines disease susceptibility, progression and protection. Finally, we evaluate current vaccine platforms and alternative control approaches, identify major knowledge gaps, and highlight future directions for the development of durable, broadly protective and sustainable intervention strategies.

## 1 Introduction

*Riemerella anatipestifer* (RA) is a gram-negative, non-motile, non-spore forming and rod-shaped bacterium of family Flavobacteriaceae that poses a severe and escalating threat to the global poultry industry (1–5). Several taxonomic revisions have been made since RA was first described. Hendrickson and Hilbert (6) initially described it as *Pfeifferella anatipestifer*. It was then classified as *Pasteurella anatipestifer* and based on phenotypic characteristics, it was later reassigned to *Moraxella anatipestifer* by Bruner and Fabricant (7). Subsequently, it was Segers et al. (1) who established the novel genus *Riemerella* based on advances in phenotypic and molecular phylogenetic analyses which demonstrated that it is distinct from *Pasteurella* and *Moraxella*. Hence, the current designation is *Riemerella anatipestifer*.

This pathogen primarily targets domestic waterfowls such as ducks and geese, but has also been reported in turkeys and wild bird species and has recently emerged as a significant infectious agent in chickens, underscoring its expanding host range and increasing significance in multi-species poultry systems (8–14). RA is the causative agent of infectious serositis, also called “new duck disease,” which manifests as an acute or chronic septicemia characterized by the clinical presentation of respiratory distress, neurological dysfunction, and locomotor impairment along with pathological lesions including fibrinous pericarditis, perihepatitis, airsacculitis, or meningitis (2, 15–17).

RA causes devastating economic loss globally with high morbidity and mortality, compounded by severe weight loss, carcass condemnations at slaughter, and treatment costs (16). To date, at least 21 RA serotypes have been described in various geographic regions; however numerous isolates has been reported to remain non-serotypable or do not correspond to the established serotypes, suggesting that the true extent of antigenic diversity is likely underestimated (10, 14, 18–22). This antigenic heterogeneity severely constrains vaccine efficacy due to limited cross-protection. Compounding this challenge is the compromised therapeutic options and concerns regarding sustainable disease control due to the emergence of multidrug resistant (MDR) RA strains (13, 23).

Despite its profound veterinary and economic significance, the precise molecular mechanisms driving *R. anatipestifer* pathogenesis lack definitive resolution. Recent molecular investigations have elucidated a critical, coordinated repertoire of virulence mechanisms that facilitate host colonization, immune evasion, systemic dissemination and neuroinvasion (24–27). These include outer membrane proteins, secretion systems, complement resistance mechanisms, and iron acquisition pathways that collectively facilitate bacterial survival, tissue invasion, and persistence in the host (24, 28–30). Although progress has been made in characterizing these bacterial virulence factors, there is still a critical knowledge gap regarding the immunological aspects of the disease, specifically the host-pathogen interactions and the mechanisms underlying host protective immunity. Current immunological research indicates that RA infection triggers strong inflammatory cascades, but the specific pathways required to confer protective immunity are still poorly resolved. In particular, the relative contribution of humoral and cellular immunity, as well as the role of mucosal immune defenses at primary sites of infection, remain unclear.

Together, the antigenic diversity, antimicrobial resistance, immune evasion and incomplete understanding of host immunity underpins a central prevention paradox in RA control. This review focuses on the immunological mechanisms of RA infection and evaluates current and emerging prevention strategies, while integrating relevant epidemiological and pathobiological insights to inform the rational design of next-generation, broadly protective RA interventions.

## 2 Epidemiology and global prevalence of *Riemerella anatipestifer*

Several complementary diagnostic approaches have been used to determine RA prevalence, and the integration of these approaches has improved detection accuracy and epidemiological resolution across studies in various geographical areas (**Table 1**). Earlier techniques by phenotypic characterization of isolates based on culture and biochemical identification are still in use but generally lack specificity, necessitating molecular confirmation for higher diagnostic resolution (15, 31, 32, 35, 53, 56, 69). PCR-based assays targeting 16s rRNA, OmpA, rpoB, gyrB, and GroEL in RA are well-established diagnostic workflows used for the specific detection of RA, along with quantitative real-time PCR approaches and loop-mediated isothermal amplification (LAMP) methods for rapid and sensitive diagnostics (10, 11, 15, 33, 34, 55, 56, 62, 69–71). Complementary methods such as matrix-assisted laser desorption ionization time of flight mass spectrometry (MALDI-TOF MS) enables species-level identification, while phenotypic characterization using fatty acid methyl ester (FAME) profiling and serotyping using slide agglutination or gel diffusion precipitin test (GDPT) are also performed to facilitate further RA characterization (10, 11, 44, 60, 62, 63). At the strain level, genotyping and DNA fingerprinting approaches, including Rep-PCR-based methods (i.e., enterobacterial repetitive intergenic consensus (ERIC, ERIC-PCR)), pulsed-field gel electrophoresis (PFGE), PCR-restriction fragment length polymorphism (PCR-RFLP), and plasmid profiling, are also implemented to resolve strain diversity, infer transmission dynamics and characterize population structure between outbreaks (10, 52, 61, 68). Using data generated through these diagnostic platforms, this section examines the global epidemiology of RA, including regional prevalence patterns, serotype distribution, and the host and ecological factors that shape pathogen circulation, transmission and persistence.

**Table 1:** Global epidemiological landscape of *Riemerella anatipestifer*.

Country/region	Year or study period	Host(s)	Prevalence/typing (serotype / genotype)	Findings	Reference
Australia	1973	fowl from a group of 3–6 weeks old chicken	Serotype A	Early identification of *Pasteurella anatipestifer* infection in fowls in Australia	(31)
	2021	adult captive-reared mandarin ducks	nonserotypable	Isolates were resistant to gentamicin and amikacin	(21)
	2021	ducks, chickens, unknown host origin	Serotype 1 (50.98%, 26/51), 6 (13.73%, 7/51), 8 (9.80%, 5/51), 9 (3.92%, 2/51), 13 (1.96%, 1/51), 14 (1.96%, 1/51), nonserotypable (17.65%, 9/51)	Report of RA serotypes commonly circulating in Australian duck production systems and the presence of *R. columbina* and *Riemerella*-like taxon 2 in chickens	(10)
Austria	2012	Geese	Genotype I (100%, 20/20)	Outbreaks in young geese were characterized by neurological signs, with 20-30% morbidity and 5-20% mortality alongside lesions including fibrinous pericarditis, perihepatitis, airsacculitis, synovitis and subcutaneous edema	(2)
Bangladesh	2013-2014	Ducks	61.66% (37/60 samples), serotyping NR	Confirmed outbreak in North East Bangladesh and isolates show high similarity to RA isolates from China	(32)
	NR	Ducks	40.38% (21/52 samples), serotyping NR	RA was isolated in high occurrence in oropharyngeal swabs of sick ducks, and in the liver and heart of dead ducks. Isolates were resistant to penicillin G, cefradine, streptomycin, neomycin, gentamycin, meropenem, and erythromycin	(33)
China	2010	Ducks	48.7% (149/306 samples); Serotype 1 (34.9%, 52/149), 2 (45.6%, 68/149), 10 (12.1%, 18/149), nonserotypable (7.4%, 11/149)	Development and evaluation of LAMP assay for RA detection.	(34)
	2020	Muscovy ducks	Serotype 15	RA serotype 15 caused spotted spleen in Muscovy duck and was resistant to polymyxin, gentamicin, neomycin, erythromycin, and enrofloxacin.	(35)
	1994-2021	Poultry	NR	Total of 417 RA isolates was subjected to AST and 95.45% (398/417) of isolates and showed resistant to four or more antimicrobial classes particularly to aminoglycosides, macrolides, quinolones, sulfonamides and tetracyclines	(13)
	2016-2022	Ducks	27.9% (195/700 samples); Serotype 1 (48%, 93/195), 2 (34%, 66/195), 7 (13%, 26/195), 10 (5%, 10/195)	Identification, serotyping and virulence of RA in Southern China	(36)
	2020-2022	Ducks	16.7% (171/1,020 samples); Serotype 1 (26.3%,45/171), 2 (26.3%,45/171), 4 (1.2%, 2/171), 6 (19.3%, 33/171), 7 (25.7%, 44/171), 10 (1.2%, 2/171)	Isolated strains were MDR with severe resistance recorded for gentamicin. Resistance genes showed high detection for tet X for tetracycline, ermF for macrolides and bla_TEM_ for β-lactams. *In vivo* experiment showed pathogenicity of RA to 7-day-old ducklings causing nervous symptoms and mortality rate from 58-70%.	(16)
	2022	Geese	74.6% (56/75 samples); Serotype 1 (66.1%, 37/56), 2 (16.1%, 9/56), 11 (14.3%, 8/56), 13 (3.6%, 2/56)	All isolates displayed severe resistance to kanamycin, streptomycin, gentamicin, azithromycin, sulfadimethoxine and sulfamethoxazole. Experimental infection to goslings resulted to serositis with neurological symptoms and mortality rate of 100%.	(37)
	2008-2023	Ducks	Serotype 1 (21.7%, 20/92), 2 (29.4%, 27/92), 4 (9.7%, 9/92), 6 (2.2%, 2/92), 7 (25.0%, 23/92), 10 (4.4%, 4/92), 11 (1.1%, 1/92), 13 (1.1%, 1/92), 14 (5.4%, 5/92)	All isolates were MDR with significant resistance to polymixin B, gentamycin, streptomycin, ciprofloxacin, doxycycline, tigecycline, azithromycin, and sulfonamides. Serotype 2 exhibited higher resistance to tigecycline and doxycycline compared to serotypes 1 and 7.	(23)
	2023	Ducks, geese	25.8% (74/287 samples), serotyping NR	Significant resistance of the isolates to aminoglycosides and macrolides and this resistance is closely associated to ARGs.	(38)
	2021-2024	Chickens and chick embryos	2.77% (752/27,136 samples); Serotype 1 (70.82%, 250/353), 4 (3.97%, 14/353), 6 (0.57%, 2/353), 7 (1.98%, 7/353), 10 (15.86%, 56/353), nonserotypable (2.83%, 10/353)	Significant resistance was observed to enrofloxacin, amikacin, and polymixin B. RA is rarely detected in chickens younger than 3 weeks but RA is predominantly isolated in chickens less than 35 weeks particularly in broiler over 30 days and 50–100 days in layer hens. RA is detected in early stage of chick embryos but it was not isolated in semen and ovarian follicles.	(14)
	2023-2024	Ducks, Chickens	90.9% (80/88 samples); Serotype 1 (19.3%, 17/88), 2 (13.6%, 12/88), 5 (35.2%, 31/88), 6 (3.4%, 3/88), 15 (17.0%, 15/88), 10 (2.3%, 2/88)	All isolates were multidrug-resistant and predominantly associated with the polymixin B, amikacin, ciprofloxacin, florfenicol and doxycycline.	(39)
	2024	Chickens	30.93% (120/388 suspected cases)	RA positive cases were primarily from layers (40.8%), broiler breeders (29.2%), broilers (22.5%) and layer breeders (6.7%). Serotype 1 was isolated and pathogenicity in mortality reached to 10% and 60% in chickens and ducks respectively during 7-days observation period.	(5)
	2024	Laying hens	NR	RA caused drop of 5-15% in egg production rate, decrease in egg quality manifested by thin-shelled eggs and sandy shelled egg, increase mortality and culling rate by 2–3 times, reduced fertilization of breeder eggs by 8-12%, increased rate of dead embryos in hatcheries (10-15%), increased proportion of weak chicks in hatched chicks (over 20%), and caused symptoms of peritonitis in weak chicks within 7 days of age	(40)
	2024-2025	Chickens	24.94% (730/2,927); Serotype 1 (95.16%, 59/62), 10 (3.23%, 2/62), nonserotypable (1.61%, 1/62)	Positive rate increased with age (6-week-old-chickens, 57.75%). Highest detection rate in chickens with joint/leg disorders (41.77%). All isolates were MDR and resistance genes ermF and tet(X) were prevalent. Virulence genes AS87_04050, fur, tbdR1, ompA were present in all isolates, *sip* was rare and exclusive to serotype 10.	(41)
Croatia	NR	Turkey	Serotype 6 (15%, 3/20)	RA was isolated from air sacs, lungs and spleens and isolates were resistant to flumequine and lincomycin.	(9)
Denmark	1976-1980	Ducks	Serotype 1 (63%), 2 (1%), 3 (15%), 8 (11%), 9 (7%), and serotype 12, rough and nonserotypable also detected at low incidence	More than one serotype can occur in the same farm and serotype detected in single farm can change from year to year.	(42)
	1980-1989	Ducks, geese	Serotype 1 (46.88%, 488/1041), 2 (0.19%, 2/1041), 3 (11.53%, 120/1041), 8 (7.20%, 75/1041), 9 (26.90%, 280/1041), 12 (0.1%, 1/1041), 13 (0.2%, 2/1041), rough (0.60%, 6/1041), nonserotypable (6.44%, 67/1041)	RA was isolated to 66.6% (983/1,477) of birds presented with salphingitis cases. Reduced egg production in flock of parents affected with RA infection	(43)
	1996	Pekin ducks	Serotype 1 (2.44%, 1/41), 4 (58.54%, 24/41), 17 (4.88%, 2/41), nonserotypable (34.15%, 14/41)	RA was present in the upper respiratory tract of healthy ducks. FAME composition of RA field strains and nonserotypable RA-like strains	(44)
Egypt	2014	Ducks and ducklings	16.7% (20/120 samples)	Higher prevalence rate in ducks (11.7%, 14/69 samples) than in ducklings (5%, 6/51 samples)	(45)
	2017-2018	Ducklings	11.67% (7/60 samples); serotyping NR	RA isolates were resistant to penicillin, ampicillin, gentamicin, streptomycin, trimehoprim/sulfamethoxazole, cefoperazone, ceftazidime, and cefepime. Isolates were MDR. Virulence genes ompA, prtC, and hagA were all identified on all isolates while sspA were detected to 5 isolates.	(46)
	2017-2018	Muscovy, pekin and mallard	8% (4/50 samples), Serotype 1	Farm history included morbidity rate from 35-60% and mortality rate at 8-20% specially at ducklings (1–4 weeks old).	(47)
	2021-2022	Muscovy, pekin and mallard	12.5% (13/104 samples); serotyping NR	RA detection was proportionally higher in mallard ducks compared to pekin and muscovy, increased with age (12–21 days vs 2–10 days), and showed slight increase incidence in winter compared to autumn.	(48)
Germany	2015-2016	Egyptian geese	70.3% (104/148 samples)	Egyptian geese were frequent carriers of RA and significant difference of RA prevalence between years of sampling.	(49)
Greece	2019	Chickens	NR	Outbreak with morbidity of approximately 10% and 5% mortality in the farm. Birds sent for necropsy showed airsacculitis, serositis, pericarditis, perihepatitis and edematous swelling around the tibio-tarsal joints. MALDI-TOF MS identified RA isolates and AST showed resistance to gentamicin, tylosin, tetracyclin, colistin sulphate, spectinomycin, lincomycin and oxytetracycline.	(50)
Hungary	2000-2014	Ducks, geese	NR	A total of 185 RA was isolated and resistance was observed to flumequine (94%), tetracycline (91.4%), erythromycin (75.1%) and streptomycin (71.4%).	(51)
	2000-2017	Ducks, geese	Serotype 1 (64.5%, 107/166), 2 (7.2%, 12/166), 4 (3.6%, 6/166), 7 (4.8%, 8/166), 10 (1.2%, 2/166), 13 (0.6%, 1/166), 17 (0.6%, 1/166), 18 (0.6%, 1/166), mixed 1 and 7 (16.9%, 28/166)	Identification of RA serotypes using ERIC-PCR.	(52)
India	NR	Ducks	NR	Cultural, biochemical and 16S rRNA diagnosis of RA in suspected field outbreak.	(15)
	2013	Ducks	NR	Heavy mortality in ducklings age 4–8 weeks. Isolates were characterized by phenotypic characterization, pathogenicity test and PCR-based method. Isolates were resistant to chloramphenicol, amoxycillin, and co-trimoxazole.	(53)
	NR	Ducks, geese	NR	Biochemical characteristics of RA isolates from Kerala and all isolates showed resistance to methicillin, metronidazole, oxacillin, penicillin G, polymyxin B, erythromycin and sulphadiazine.	(54)
	2019	Ducks	13.3% (13/98 samples); serotyping NR	Heavy mortality recorded at 4–8 weeks of age with signs including respiratory and neurological signs such as trembling of head and neck, paddling of legs and ataxia. Isolates were resistant to gentamicin and cefazolin. PCR-based confirmation using 16s rRNA and *gyrB.*	(55)
	2019-2020	Ducks	36.84% (28/76 tested samples)	Identification by phenotypic characteristics and PCR-based assay targeting 16S rRNA and ERIC sequence. Epidemiological data of farm outbreak included overall mortality of 53% and morbidity of 39% in affected flocks. Mortality of 57% was recorded in ducklings and 19% in adult ducks. Highest detection in brain, spleen and ocular swabs samples. 78% (28/36)RA PCR positive detection among phenotypically indentified samples.	(56)
	NR	Ducks	31.79% (144/453 samples); serotyping NR	Mortality and morbidity observed in ducklings less than 5 weeks of age. Death occurred within short period after clinical manifestation of greenish-white diarrhea, off fed, torticollis, tremor of head and neck and incoordination of movement.	(57)
Japan	2014	Ducks	RA isolated in pure culture in organs of samples birds (2/4)	Farm history showed mortality rate of 16% (316/2,020 birds). RA isolates were resistant to ampicillin, benzylpenicillin, cloxacillin, enrofloxacin, kanamycin and norfloxacin.	(58)
Poland	2015-2021	Chickens, turkeys, ducks, geese	NR	Identification of RA isolates using MALDI-TOF MS and PCR. Isolates have high MIC_50_ and MIC_90_ for gentamycin, amikacin and colistin. Frequent ARGs identified were *tet(X)* and *ermF*.	(11)
	2020-2022	Ducks, geese, turkeys, white-fronted geese, greylag geese, Taiga bean geese	Commercial poultry (19%, 24/126 samples: 35.7% in ducks, 30% in geese, 3.2% in turkeys); Wild geese (94.7%, 18/19 samples); serotyping NR	RA isolated in commercial poultry flocks and and wild birds in Poland.	(12)
Singapore	1982-1990	Ducks	Serotype 1 (32.7%, 115/352), 2 (4.8%, 17/352), 6 (2.3%, 8/352), 7 (8.5%, 30/352), 10 (14.8%, 52/352), 11 (3.4%, 12/352), 14 (3.6%, 13/352), 15 (25.6%, 90/352), 16 (0.3%, 1/352), 17 (2%, 7/352), P (0.3%, 1/352), nonserotypable (1.4%, 5/352), rough (0.3%, 1/352)	Serological typing of duck isolates. Identification and designation of serotypes 17, 18, and 19, and replacement of serotype 4 reference.	(59)
South Korea	2011-2012	Wild birds	Pharynx (69.6%, 71/102 samples), Cloacal swab (2%, 19/944 samples); Serotype 1, 4, and 7	All isolates (n=33) were resistant to at least one antibiotic and most prevalent resistance was for kanamycin, amikacin, neomycin and streptomycin.	(8)
	2002-2015	Ducks	Serotype 1 (15.3%, 20/130), 2 (8.9%, (12/130), 5 (0.7%, 1/130), 6 (5.2%, 7/130), 7 (0.7%, 1/130), 11 (11.9%, 16/130),13 (7.4%, 10/130), 14 (2.3%, 3/130), 17 (0.7%, 1/130), 19 (3.7%, 5/130), nonserotypable (17.7%, 23/130), reacted to 2 or more antiserum (23.85%, 31/130)	Serotypes 1, 2, 5, 6, 7, and 11 were associated with clinical isolates while serotypes 13, 14, 17, 18, 19 and 21 were only found in pharyngeal isolates. Systemic strains were more resistant to bactericidal effect of serum compared to strains isolated from the phraynx.	(60)
Taiwan	NR	Pekin ducks, Roman geese	Serotype 2 (44.44%, 4/9), 4 (11.11%, 1/9), 5 (11.11%, 1/9), 6 (33.33%, 3/9)	Marked genotypic and serotypic heterogeneity was observed across and within farms, including coexistence of multiple variants within a single host	(61)
	2009-2010	Ducks, geese	Serotype 1 (2.6%, 2/76), 2 (9.2%, 7/76), 3 (18.4%, 14/76), 4 (6.6%, 5/76), 5 (1.3%, 1/76), 6 (10.5%, 8/76), 8 (13.2%, 10/76), 9 (11.8%, 9/76), 10 (1.3%, 1/76), 11 (11.8%, (9/76), 14 (9.2%, 7/76), 15 (5.3%, 4/76), 17 (1.3%, 1/76), 19 (1.3%, 1/76), 20 (7.9%, 6/76), 21 (32.9%, 25/76), B (39.5%, 30/76), Unknown/nonserotypable (14.5%, 11/76)	All isolates were resistant to colistin and more than half of the isolates had resistace to amikacin, gentamicin, nalidixic acid and streptomycin. RA was isolated from slaughter house and asymptomatic waterfowl.	(20)
	2014	Pekin and muscovy ducks	55.6% (15/27 isolates); Serotype 1 (9.1%, 1/11), 4 (9.1%, 1/11), 6 (9.1%, 1/11), 17 (9.1%, 1/11), 19 (9.1%, 1/11), nonserotypable/unidentified (54.55%, 6/11)	Identification using 16S rRNA and OmpA PCR-based assay and serotyping using GDPT.	(62)
Thailand	1988-1989	Ducks	Serotype 1 (60%, 12/20), 6 (5%, 1/20), nonserotypable (35%, 7/20)	Serotyping was performed by GDPT. Isolates were resistant to colistin, gentamicin, kanamycin and sulfadimethoxin.	(63)
	1994-1999	Ducks	Serotype 1 (5%, 4/80), 3 (1.25%, 1/80), 5 (6.25%, 5/80), 6 (2.5%, 2/80), 7 (22.50%, 18/80), 8 (2.5%, 2/80), 10 (6.25%, 5/80), 11 (2.5%, 2/80), 13 (3.75%, 3/80), 14 (6.25%, 5/80), 15 (5%, 4/80), 17 (1.25%, 1/80), 18 (2.5%, 2/80), 19 (3.75%, 3/80), 21 (5%, 4/80), reacted to more than 2 antiserum for serotype (20%, 16/80), reacted to more than 3 antiserum serotype (2.5%, 2/80), untypable strain 698/95 (1.25%, 1/80)	20% morbidity and 18% mortality. Proposed new reference for serotype 20. Serotypes 4, 9, 12 and 16 not detected.	(64)
	2021-2023	Ducks, Chickens	Ducks: Serotype 1 (5.41%, 2/37), 5 (5.41%, 2/37), 7 (18.92%, 7/37), 10 (10.81%, 4/37), 11 (5.41%, 2/37), 17 (2.7%, 1/37), nonserotypable (51.35%, 19/37); Chickens: Serotype 1 (100%, 10/10)	All bacterial isolates tested have high resistance to colistin, and most prevalent resistant genes were tet(X2) and lnu(I).	(65)
United States	1975-1979	Ducks	Serotype 1 (24-56.9%), 2 (23-53.9%), 3 (0.4-4.2%), 5 (13.1-21.7%), 7 (0.2-1%), 8 (0-0.3%)	More than 95% of the isolates were from serotypes 1, 2, and 5. Serotypes 2 and 5 showed reciprocal cross-reactivity.	(66)
	1986	Turkey	Serotype 1	Respiratory signs and increased morbidiy and mortality starting 6–15 weeks of age. Total mortality 5-46%.	(67)
	NR	Pekin Ducks	Serotype 5 (28.57%, 4/14), nonserotypable (71.43%, 10/14)	Identification was performed using serotyping and DNA fingerprinting. Affected ducks showed lateral recumbency accompanied by paddling and opisthotonus. Necropsy findings revealed fibrinous polyserositis, epicarditis, pericarditis, perihepatits, airsacculitis, bronchopenumonia, and pleuritis.	(68)
Vietnam	2018-2020	Ducks	16.91% (69/408); Serotype 1 (4.3%, 3/69), 6 (1.4%, 1/69), 8 (2.9%, 2/69), 10 (31.9%, 22/69), 20 (2.9%, 2/69), nonserotypable (56.5%, 39/69)	Over 40% of the isolates were resistant to nalidixic acid, streptomycin, norfloxacin, kanamycin, oxacilin, and getamicin.	(22)
	2024	Muscovy ducks	38.24 % (26/68), serotyping NR	RA was detected in blood, heart, liver, spleen and lungs. Isolates were resistant to enrofloxacin and ampicillin. Isolates also demonstrated multidrug resistance patterns.	(17)

ARG, antibiotic resistant genes; ERIC-PCR, enterobacterial repetitive intergenic concensus-polymerase chain reaction; FAME, fatty acid methyl esters; GDPT, gel-diffusion precipitin test; MALDI-TOF MS,matrix assisted laser desorption ionisation time of flight mass spectrometry; MDR, multi-drug resistane; NR, not reported in the original study; RA, *Riemerella anatipestifer.*

### 2.1 Global distribution, prevalence and serotype diversity of *Riemerella anatipestifer*

The reported RA prevalence varies substantially between locations and ranges from 2.7–24.94% detection rates in recent large-scale surveillance studies to approximately 30-90% in outbreak investigations (5, 14, 16, 17, 32–34, 38, 39, 41, 49, 56, 57, 62). The majority of available epidemiological data originated from Asia, including Bangladesh, China, India, Japan, Singapore, South Korea, Taiwan, Thailand and Vietnam. In waterfowls, prevalence ranges from moderate to high outbreak-associated detection, including 40.38-61.66% in Bangladesh, 16.7-48.7% in China, 13.3-36.84% in India, 16.91-38.24% in Vietnam, and 55.6% in Taiwan (16, 17, 22, 32–34, 36, 38, 55–57, 62). A report from Japan further confirmed the presence of RA in duck production systems, although recent epidemiological data remain limited (58). In China, large-scale surveillance in chickens indicates low overall detection (2.77-24.94%), although targeted investigations report higher positivity (30.93%), particularly in older broilers, layers, and breeder flocks (5, 14). RA has extensive serotype heterogeneity across Asia, as reported in clinical and field isolates, with serotypes 1, 2, 5, 6, 7, and 10 most consistently detected (14, 16, 20, 22, 23, 34, 36, 39, 59–65). A substantial proportion of RA isolates also remain non-serotypable, and occasional multi-sera cross-reactivity during agglutination-based serotyping classification, underscoring the inherent limitations of the current serotype classification of RA (14, 20, 22, 34, 59, 60, 62–65).

Outside Asia, RA has been reported on other continents including Europe (i.e., Austria, Croatia, Denmark, Germany, Greece, Hungary, Poland), North America (i.e., United States) Oceania (i.e., Australia) and Africa (i.e., Egypt), although data are still proportionately constrained. In Europe, marked host-associated variation is evident with a high prevalence reported in Egyptian geese (70.3%) in Germany and host-stratified prevalence in Poland, ranging from 35.7% in ducks, 30% in geese and 3.2% in turkeys in commercial flocks to 94.7% in wild geese (12, 49). Earlier reports in Denmark reveal pronounced serotype diversity and temporal turnover in farms, with serotypes 1, 3, 4, 8 and 9 predominating along with non-serotypable variants (42–44). There is comparable serotype heterogeneity in Hungary, where serotypes 1, 2, 4, 7, 10, 13, 17, and 18, as well as mixed infections in waterfowl populations have also been reported, whereas outbreaks in Austria were associated with a single dominant genotype (2, 52). Beyond waterfowl, RA was also detected in chickens in Greece and RA serotype 6 in turkeys in Croatia (9, 50). In the United States, isolates are predominantly from ducks with serotypes 1, 2, 5 and non-serotypable account for most isolates, while additional serotypes occur sporadically, and there is the detection of serotype 1 in turkeys (66–68). In Australia, RA circulates in both chickens and ducks, with serotypes 1 and non-serotypable isolates predominating along with serotypes 6, 8, 9, 13 and 14 (10, 21, 31). Lastly, data in Africa are largely limited to Egypt, where moderate prevalence (8-12.5%) has been reported from outbreak investigations in waterfowl populations with variations between host species and age groups, along with the identification of serotype 1 (45–48).

### 2.2 Epidemiological determinants and transmission dynamics of *Riemerella anatipestifer*

RA epidemiology is shaped by an interplay of host, environmental and population-level factors that collectively influence transmission dynamics and detection patterns. Susceptibility is consistently reported to be higher in waterfowl, particularly in ducks, with breed-associated differences, as evidenced by higher detection in mallard ducks compared to pekin and Muscovy ducks (12, 39, 48). Age-related patterns have also been documented, although findings vary between studies and most reports indicate a higher prevalence in younger ducks during the early post-hatch period, increased detection in birds aged 2–10 days compared to 12–21 days, and severe mortality extending up to 4–8 weeks of age, suggesting sustained susceptibility throughout the juvenile stage (45, 47, 48, 53, 55–57). In contrast, RA is rarely detected in very young chickens (<3 weeks) but is more frequently identified in older birds, particularly in broilers beyond 30 days, laying hens at 50–100 days, and turkeys between 6–15 weeks of age, indicating species- and stage-dependent susceptibility across different production systems (14, 67) (**Table 2**). RA has also been detected during early embryonic stages in chicken; however, the absence of isolation in semen and ovarian follicles suggests that the role of direct vertical transmission remains unclear (14). At the population level, previous reports have noted pronounced genotypic and serotypic heterogeneity, with multiple serotypes co-circulating in individual farms, temporal turnover of dominant serotypes across production cycles, and the coexistence of multiple variants in a single host (42, 61). Available evidence further suggests that detection may vary according to sampling strategy, and high recovery was reported from blood, brain, spleen, liver, heart, air sacs and non-cloacal sampling sites, including ocular swabs and pharyngeal swabs, compared to cloacal swabs (8, 9, 17, 33). In addition, RA has been isolated from asymptomatic birds, waterfowls in slaughterhouses, and the upper respiratory tract of clinically healthy ducks, supporting the possibility of subclinical colonization and persistence in host populations (20, 44). Seasonal variation appears modest, although a report from Egypt suggests slightly higher detection during cooler periods, potentially reflecting how environmental persistence or management-related factors influence exposure dynamics (49). In addition, temporal variations in prevalence between sampling years further supports the dynamic and fluctuating nature of RA circulation in affected populations (48).

**Table 2:** Comparative host susceptibility, affected age or production stage and immune characteristics associated with *R. anatipestifer* infection.

Host species/breed	Relative susceptibility to RA	Reported age/stage of affected birds	Key immune characteristics	Reference/s
Waterfowl (ducks)	Highest susceptibility among avian hosts with breed-associated differences reported	Early post-hatch to juvenile stage, high detection at 2–10 days vs 12–21 days, severe mortality up to 4–8 weeks	Sustained inflammatory responses and IL-17-associated immunopathology	(12, 39, 48, 72, 73)
Mallard ducks	Higher detection and susceptibility than Pekin and Muscovy ducks	NR	Higher detection rates compared with Pekin and Muscovy ducks	12, 39, 48)
Pekin ducks	Lower than mallards	NR	NR	
Muscovy ducks	Lower than mallards	NR	Causes spotted spleen disease	(17, 35, 48)
Ji’an Red-feathered ducks	Relatively more resistant	NR	Resistant birds-maintained antibody profiles comparable to healthy controls while susceptible birds showed reduced IgA with stable IgG and IgM.	(74)
White Kaiya ducks	Relatively more susceptible	NR	Resistant birds had stable IgA and moderate increase in IgM while susceptible birds also have stable IgA but increased IgG and IgM, and significantly reduced IL-17A.	(74)
Chickens	Relatively resistant	Rarely detected <3weeks of age, more frequently detected in broilers (>30 days) and laying hens (50–100 days)	Balanced Th1/Th2 immune responses and controlled inflammatory signaling with effective antimicrobial response.	(5, 41, 50, 72, 75)
Geese	Susceptible	NR	In comparison to ducks, they shared the classical septicemic-serosal disease pattern.	(2, 37)
Turkeys	Susceptible	Primarily reported at 6–15 weeks of age	Development of respiratory disease and fibrinous serositis. Robust systemic RA-specific antibodies post-respiratory inoculation.	(67, 76)

NR, not reported in the cited literature.

Along with its complex epidemiology, RA increasingly exhibits multidrug-resistant (MDR) phenotypes that further complicate disease control and outbreak management. Reduced susceptibility against β-lactams, tetracyclines, sulfonamides, aminoglycosides, quinolones, and macrolides often associated with resistance determinants including *bla*, *tet*, *erm*, *lnu*, *sul, ran, floR* and *qnr* genes has been commonly reported, highlighting constraints on therapeutic efficacy and the need for continued surveillance in poultry production systems (11, 13, 14, 16, 20, 23, 33, 37, 38, 41, 46, 51, 54, 58, 65, 77, 78). Despite the global distribution of RA, the current understanding of its epidemiology is still uneven due to geographical bias in available reports, limited data from several regions including Latin America and parts of Africa, and inconsistencies in diagnostic and serotyping approaches that undermine interpretation across studies. Moreover, the high degree of serotypic and genotypic heterogeneity, together with the occurrence of non-serotypable and cross-reactive isolates, continues to challenge the identification of conserved antigenic targets and predictability of protective immune responses. These limitations collectively underscore the need for a deeper understanding of RA immunological landscape and host-pathogen interactions to support the development of broadly protective vaccines and more effective prevention strategies.

## 3 Pathogenesis and pathological manifestations of RA infection

**Figure 1:**
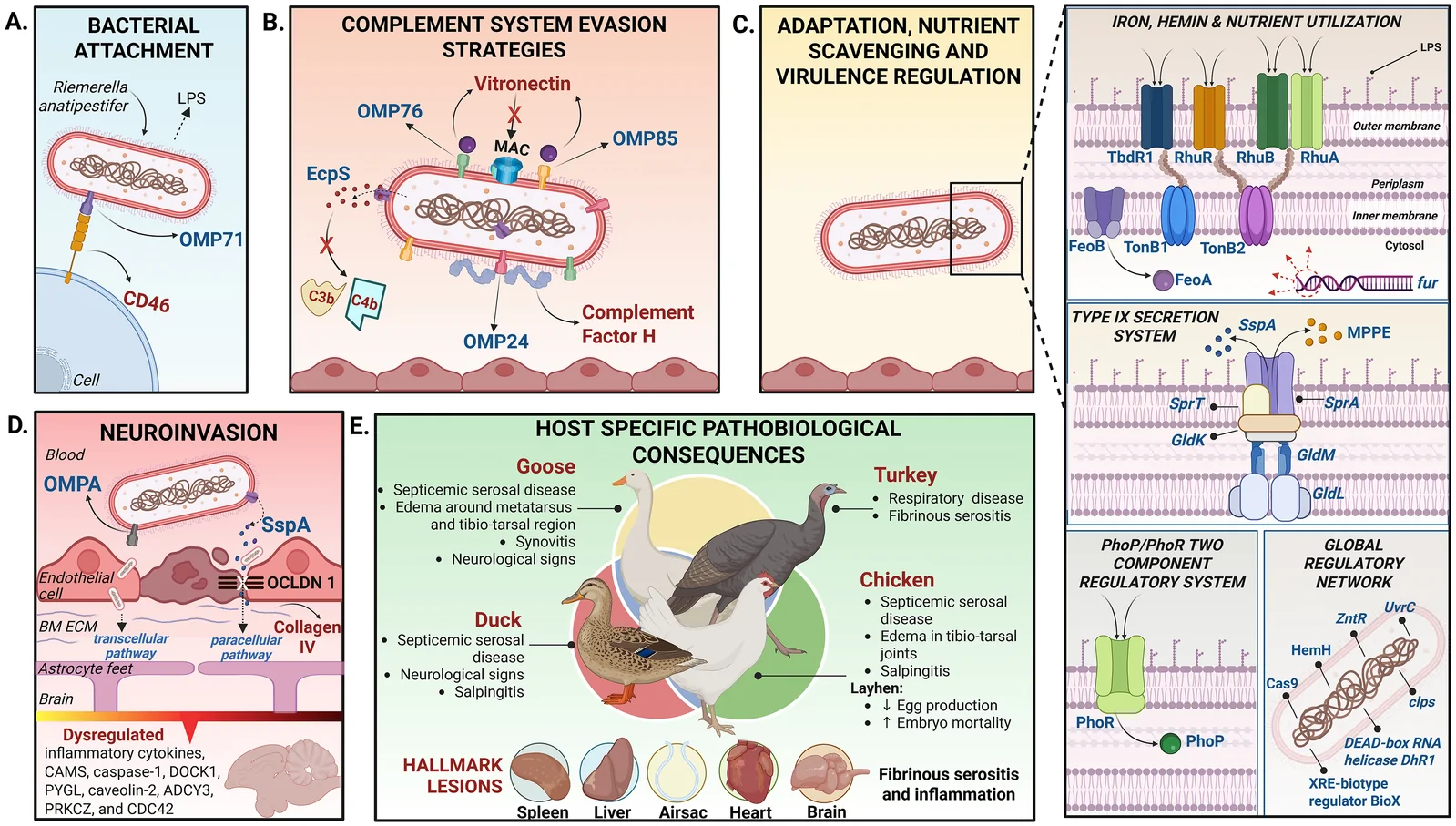
Molecular mechanisms underlying *Riemerella anatipestifer* pathogenesis, immune evasion, and disease outcomes. The schematic summarizes the current evidence supporting the molecular determinants of *R. anatipestifer* (RA) colonization, immune evasion, nutrient acquisition, systemic dissemination, and tissue injury. **(A)** Following host entry, RA adheres to cells through engagement of outer membrane proteins (OMP) to host receptors to facilitate early colonization, such as OMP71-mediated interaction with CD46. **(B)** RA subsequently evades complement-mediated killing through complementary mechanisms targeting distinct stages of the complement cascade. OMP85 and OMP76 recruit host vitronectin to inhibit membrane attack complex (MAC) formation, OMP24 recruits complement factor H to suppress the alternative pathway, and secreted extracellular protease S (EcpS) degrades C3b and C4b. By collectively impairing complement activation, opsonization, cascade amplification and terminal MAC formation, these mechanisms promote serum resistance and bacterial survival. **(C)** During systemic dissemination, RA counters host nutritional immunity through ferric uptake regulator (*fur*)-mediated adaptive regulation of TonB- and Feo-system. Fur dynamically regulates these systems to enhance uptake under iron-limited conditions while restricting excess accumulation to maintain homeostasis and limit oxidative stress. Concurrently, the Type IX Secretion system (*SprA, SprT, GldK, GldM, GldL*) secretes virulence effectors such as subtilisin-like serine protease (*SspA*) and metallophosphoesterase (MPPE), whereas the PhoP/PhoR two component systems and other global regulators (UvrC, ZntR, HemH, Cas9, clps, DEAD-box RNA helicase DhR1, XRE-biotype regulator BioX) coordinate stress adaptation and virulence for bacterial persistence and fitness. **(D)** Systemic dissemination enables RA to invade central nervous system and neuroinvasion through transcellular and paracellular pathway mediated by OmpA-dependent endothelial invasion and *SspA*-mediated degradation of occludin 1 (OCLN 1) and collagen IV, which results to blood brain barrier disruption and dysregulated expression of proteins and cytokines associated to cytoskeletal remodeling, endothelial function, inflammatory signaling and vascular injury. **(E)** Collectively, these virulence mechanisms culminate in host-specific disease manifestations, including septicemic fibrinous polyserositis, respiratory disease, neurological signs, synovitis, salpingitis, reduced egg production and increased embryo mortality with characteristic fibrinous serositis lesions (spleen, liver, airsacs, and heart) and inflammation (brain). BM ECM, basal membrane extracellular matrix; LPS, lipopolysaccharide; OMP, Outer Membrane Protein. All the bacterial determinants are in blue text and the illustration is created using Biorender (https://BioRender.com/q6gvyku).

The pathogenesis of RA infection is increasingly recognized as a multistage process beginning with bacterial entry into the host and initial interactions with host cells, followed by within-host adaptation, septicemic dissemination, invasion and colonization of affected organs, and, in severe cases, neuroinvasion. These interconnected events are driven by bacterial surface structures, complement-evasion strategies, nutrient-acquisition systems, the type IX secretion system and its secreted effectors, and global regulatory networks. Collectively, these determinants enable immune evasion, virulence, adaptation to host-imposed nutritional and environmental stresses, and bacterial survival and persistence. These pathogen-mediated processes culminate in characteristic gross and microscopic lesions and diverse clinical manifestations in infected hosts. This section examines these bacterial determinants and pathogenic events underlying RA infection, together with resulting pathologies across avian hosts (**Figure 1**; **Table 3**).

**Table 3:** Summary of *R. anatipestifer* virulence-associated determinants and their contributions to the progression of RA infection.

Major bacterial determinants	Mechanism or principal function	Pathogenic outcome	References
Outer Membrane Protein A (OMPA)	Role in epithelial cell adhesion and invasion, virulence-associated, invasion in DBMEC	Stable colonization, invasion and progression of infection	(27, 28)
OMPH	Role in adhesion and invasion (DEF cells), virulence associated		(80)
OMP71	Promotes bacterial attachment and virulence by interaction with CD46		(81)
PorV and PosF	Role in adhesion and invasion (DEF cells)		(82)
OMP76 and OMP85	Recruitment of vitronectin to inhibit membrane attack complex (MAC)	Reduced complement-mediated killing and phagocytic clearance promoting bacterial survival in blood and septicemic dissemination	(26, 83)
OMP24	Recruitment of host complement factor H to inhibit the complement alternative pathway		(84)
Extracellular protease S (EcpS)	Block complement C3b and C4b		(24)
Feo system (FeoA, FeoB), TonB-dependent receptors (TbdR1, RhuR, RhuA, RhuB), TonB energy transducers (TonB1, TonB2), RhuH, YbiT	Acquisition of ferrous iron and hemin	Adaptation to host-imposed nutrient immunity thereby promoting bacterial fitness, colonization, persistence and virulence	(29, 30, 85–91)
Ferric uptake regulator (fur)	Regulation of iron/hemin acquisition, iron homeostasis and oxidative-stress resistance		(92–94)
Type IX Secretion System (T9SS): *GldG*, *GldK*, *GldL*, *GldM*, *SprA* and *SprT*	Role in gliding motility, biofilm formation, and secretion of virulence-associated effectors	Enhanced tissue invasion, bacterial persistence, dissemination and pathogenicity	(25, 95–99)
Subtilisin-like serine protease (SspA)	T9SS secreted effector that degrades tissue barrier (i.e.Occludin, Collagen IV in endothelial cells), enhanced resistance to antimicrobial peptide (i.e LL-37), inhibited monocyte chemotaxis	Increased barrier permeability, invasion, systemic dissemination, and blood-brain barrier (BBB) collapse leading to neuroinvasion	(100–102)
Metallophosphoesterase (MPPE)	T9SS secreted effector with phosphatase activity and may play a role in removing reactive oxygen intermediates, virulence-associated effector	Extracellular survival and systemic dissemination	(103)
PhoP/PhoR two-component signaling system	Signal transduction and global regulation of virulence-, stress adaptation-, and metabolism-associated genes	Promoted host adaptation, systemic persistence and virulence	(104, 105)
Excinuclease UvrC	UvrABC subunit that mediate excision repair, supports oxidative-stress tolerance, iron utilization, biofilm formation and adhesion and invasion		(106)
Other regulatory, metabolic, and stress-adaptation determinants	DEAD-box RNA helicase DhR1, XRE-type transcriptional regulator BioX, Cas9: Adaptive control of RNA processing and gene expression; hemH: heme biosynthesis, zntR: zinc homeostasis, clps: stress response; NuoB and Sdhc: respiratory function, antibiotic susceptibility, oxidative stress tolerance, virulence; MoxR-*bat*: stress adaptation; TerC: manganese homeostasis	Maintains metabolic fitness and survival under host-imposed stress to promote tissue colonization, persistence and virulence	(107–115)

DBMEC, duck brain microvascular endothelial cells; DEF, Duck Embryo Fibroblast; RhuA, *R. anatipestifer* hemin uptake receptor A; RhuB, *R. anatipestifer* hemin uptake receptor B; RhuR, *R. anatipestifer* hemin uptake receptor R.

### 3.1 Mucosal colonization, adhesion and immune evasion

Infection is primarily initiated by horizontal transmission via the respiratory tract or oral route, or by mechanical entry via skin abrasions and wounds (67, 76, 116). Following mucosal colonization, RA utilizes multiple outer-membrane proteins, including OmpA, OmpH, OMP71, OMP76 and OMP85, to collectively mediate host-cell adhesion and invasion while conferring serum and complement resistance, thereby facilitating progression from localized infection to acute septicemic dissemination (26–28, 60, 80, 81). More recently, PorV, an outer membrane component of the Type IX secretion system (T9SS), and PosF, a porin, were also identified as surface adhesins that promote adhesion and invasion of RA to duck embryo fibroblast, however their cognate receptor remains unknown (82). Mechanistically, OMP71 promotes bacterial attachment through interaction with the host complement regulatory receptor CD46, whereas OMP76 and OMP85 enhances serum resistance with the recruitment of host vitronectin to inhibit membrane attack complex (MAC) formation (26, 81, 83). In addition, OMP24 has been identified as a factor H-binding protein capable of recruiting host complement factor H to inhibit the complement alternative pathway and enhance bacterial survival in normal duck serum, indicating the importance of complement evasion for RA persistence (84). During septicemia, RA further limits complement-mediated clearance through secretion of extracellular protease S (EcpS), which block complement C3b and C4b via the classical and lectin pathway to reduce opsonization and phagocytic killing (24). Consistent with this, systemic isolates were found to have significantly greater resistance to serum bactericidal activity than pharyngeal isolates, highlighting the importance of immune evasion during early dissemination (60).

### 3.2 Nutritional immunity and iron acquisition

Trace metals are indispensable for the survival and maintenance of cellular homeostasis in both vertebrate hosts and bacterial pathogens. During infection, vertebrate hosts restrict microbial access to these essential micronutrients (i.e. iron) through a defense mechanism known as nutritional immunity, creating a nutrient metal-limited environment that constrains bacterial growth and survival (117). In response, bacterial pathogens have evolved specialized mechanisms to overcome this host-imposed nutrient restriction. To date, multiple potentially overlapping iron-acquisition pathways have been described in RA to facilitate nutrient scavenging in an iron-restricted host environment and support bacterial survival, systemic dissemination and virulence during infection. These include the Feo system, comprising feoA and feoB for ferrous iron transport, as well as TonB1- and TonB2-associated iron and hemin acquisition systems in which surface-associated hemin-binding proteins cooperate with TonB-dependent receptors (TbdRs) such as TbdR1, *R. anatipestifer* hemin uptake receptor (RhuR), and *R. anatipestifer* hemin uptake receptor B (RhuB) to mediate hemin uptake and nutrient utilization (29, 30, 85–89).

Early transcriptomic analysis of RA in iron limited conditions revealed selective remodeling of this acquisition network, wherein feoA-feoB, a putative FepA-HmuY-like hemin acquisition system, and several Ton-B dependent receptors (B739_0094, B739_0103, B739_0173, B739_1068, and B739_1416) predicted to participate in iron or hemin uptake were induced, while other predicted TonB-dependent transporters (B739_0115, B739_0876, B739_1045, B739_1343, B739_0216, B739_0329, and B739_0389) were downregulated. Interestingly, the transcription of *tonB* genes in RA is not significantly changed in iron-limited conditions, suggesting that RA adapts primarily by differentially regulating substrate-specific receptors and hemin-binding components rather than uniformly activating the entire TonB energy-transduction machinery (93). More recent receptor-specific studies further delineated a specialized and partially redundant hemin-acquisition network in RA. RhuA, a surface-exposed outer membrane protein, were reported to bind hemin and cooperates with TonB2-dependent receptor RhuR to facilitate the utilization of hemin from duck hemoglobin (30). Subsequent work also identified RhuB as a second, highly conserved TonB2-dependent receptor that directly binds hemin and supports its acquisition from duck hemoglobin, with genetic evidence indicating that RhuA also cooperate with RhuB. Nevertheless, deletion of RhuB did not attenuate RA *in vivo*, suggesting that parallel hemin-acquisition pathways compensate for the loss of an individual receptor and preserve bacterial fitness and virulence (89). Likewise, RhuH was identified as an outer membrane vesicle associated hemophore that extracts heme from duck hemoglobin and supports heme-dependent growth (90). *YbiT*, an ATP-binding cassette (ABC) transporter, was also shown to contribute to iron acquisition, stress adaptation and RA virulence (91). Collectively, these findings reveal an expanding and partly redundant iron- and hemin acquisition network in RA that support bacterial fitness.

Central to this adaptive response is the ferric uptake regulator (*fur*), a global iron-responsive transcriptional regulator that coordinates bacterial adaptation to host-imposed nutritional immunity. When intracellular iron is abundant, iron-bound *fur* suppresses high affinity-iron acquisition systems, preventing toxic iron accumulation and oxidative damage. Conversely, when iron becomes scarce, *fur*-dependent repression is relieved, enabling the coordinated expression of iron-uptake pathways that promote RA survival and persistence within the iron-restricted host environment (92, 93, 118). Transcriptomic analyses demonstrated that *fur* regulates broad regulatory network encompassing genes involved in iron acquisition and transport systems, cellular metabolism, and membrane biogenesis pathways. Complementary electrophoretic mobility shift assays (EMSA) further confirmed the direct binding of *fur* to the promoter regions of fur-regulated genes such as hemin uptake receptor HmuR, Type IX secretion system component (T9SS) *sprT*, and iron transport-associated outer membrane proteins (RAYM_01847 and RAYM_09824), establishing *fur* as a global regulator of iron homeostasis in RA (92). Functional evidence further showed that loss of *fur* increased the expression of putative TonB-dependent receptors (B739_0103 and B739_0173), increased susceptibility to the iron-dependent antibiotic streptonigrin and hydrogen peroxide, accompanied by elevated intracellular reactive oxygen species (ROS) under iron-rich conditions. The *fur* mutant also exhibited reduced survival in non-inactivated duck serum, impaired tissue colonization, and marked attenuation in ducklings (118). Collectively, these findings establish *fur* as a master regulator of iron homeostasis, oxidative-stress resistance and virulence, thereby promoting RA fitness, persistence within host, and systemic pathogenicity.

### 3.3 Type IX secretion system and virulence regulation

Increasing evidence also identified the Type IX secretion system (T9SS) as a central virulence platform coordinating protein secretion, tissue invasion, environmental adaptation, and host-pathogen interactions in RA (25, 95). T9SS-associated components in RA, including *GldG*, *GldK*, *GldL*, *GldM*, *SprA* and *SprT*, are essential for the secretion of virulence-associated effectors and pathogenicity, as disruption of these genes markedly attenuates virulence by impairing protein secretion, gelatinase activity, gliding-associated motility, biofilm formation and bacterial dissemination (25, 95–99). Among secreted effectors, subtilisin-like serine protease (SspA) and metallophosphoesterase (MPPE) contribute significantly to tissue-barrier damage, monocyte chemotaxis inhibition, while enhancing resistance to antimicrobial peptides and oxidative stress, thereby supporting RA persistence and virulence (100–103). Recent comparative genomic analyses have identified additional virulence-associated determinants involved in host colonization, iron acquisition, intracellular survival and immune evasion, including brkB, ClpC, ybt, Type IV secretion system (T4SSs)-associated gene, hemolysin-associated gene, capsule-associated genes, and lipooligosaccharide (LOS)-associated genes, highlighting the multifactorial nature of RA pathogenicity and adaptation across avian hosts (119).

### 3.4 Stress adaptation and global virulence regulatory networks

Beyond these structural and secretion-associated pathways, virulence determinants are additional regulatory and stress-adaptation pathways that contribute significantly to RA pathogenicity. Notably, the global regulatory pathway PhoP/PhoR two-component signaling system has been described to modulate aerotolerance, oxidative stress adaptation, nutrient and membrane transport, and virulence-associated gene expression, whereas the excinuclease UvrC contributes to oxidative stress tolerance, iron acquisition, biofilm formation, adhesion and invasion (104–106). Additional regulatory determinants including the DEAD-box RNA helicase DhR1, XRE-type transcriptional regulator BioX, Cas9, the heme biosynthesis-associated gene hemH, the zinc homeostasis regulator zntR, and the stress response-associate gene clps have been implicated in bacterial fitness, metabolic adaptation, metal ion homeostasis, gene regulation, and virulence maintenance, showing the complexity of the regulatory networks underlying RA pathogenicity and host adaptation (107–110, 112, 113). Recent studies further link MoxR, a chaperone-like AAA+ ATPase that modulates the *Bat* operon, and TerC, a membrane manganese exporter, to stress adaptation and pathogen fitness in RA (111, 114). Likewise, NuoB, and SdhC, subunits of respiratory complexes I and II, respectively, further connect respiratory function to antibiotic susceptibility and oxidative-stress tolerance (115). However, the interplay and conservation of these mechanisms across strains remain unresolved.

### 3.5 Systemic dissemination and neuroinvasion

Following the establishment of bacteremia, RA rapidly disseminates to highly vascularized serosal and reticuloendothelial tissues and produces hallmark lesions of fibrinous polyserositis, pericarditis, perihepatitis, airsacculitis, splenic necrosis, meningitis or multisystemic inflammation injury (17, 48, 68, 74, 120, 121). Disease progression is particularly severe in young ducklings, where outbreaks are frequently associated with acute septicemia, neurological manifestations, and can cause morbidity from 35-60% and a mortality rate from 8-70% depending on the outbreak (16, 47, 56–58). Experimental infection studies in ducks further demonstrated a mortality rates of 10-80% depending on the serotype (5, 72). Clinically affected ducks first exhibit anorexia, an off-fed, mucous discharge from the mouth and nostrils, and respiratory distress before advancing to severe neurological dysfunction characterized by tremors of the head and neck, torticollis, ataxia, paddling movements, lateral recumbency, and incoordination reflecting the pronounced neurotropic nature of advanced RA infection (48, 55, 57, 68).

Neuroinvasion represents a defining feature of advanced RA infection, where the pathogen breaches the blood-brain barrier (BBB) through coordinated transcellular and paracellular invasion mechanisms culminating in suppurative meningitis, severe neurological dysfunction, and rapid neurological deterioration (27, 101, 122). *In vitro* models using brain microvascular endothelial cells (BMECs) are well-established platforms to resolve bacterial BBB invasion (123–128). A recent development and characterization of species-specific duck BMECs has elucidated the role of OmpA as a critical transcellular determinant. Within this species-specific model, OmpA proved dispensable for initial attachment but indispensable in endothelial cell invasion, particularly the OmpA extracellular domain 3 loop (amino acids 230-242) and the truncated isoform OmpA1164, (amino acids 102-488), establishing OmpA as a major mediator of invasion (27). Complementing this intracellular route, RA also crosses the BBB through a paracellular pathway mediated T9SS-exported effector SspA. Rather than traversing endothelial cells, SspA acts as direct extracellular proteolytic effector that hydrolyzes the extracellular domains of the tight junction protein occludin (OCLN) between adjacent endothelial cells, and cleaves collagen IV within the endothelial basement membrane. This targeted proteolysis results in a breach in the neurovascular barrier seals, allowing paracellular bacterial entry into the central nervous system (CNS). Subsequently, this triggers downstream upregulation of host inflammatory cytokines and cell adhesion molecules (CAMs) such as VCAM-1 (vascular endothelial cell adhesion molecule 1), PECAM-1 (platelet endothelial cell adhesion molecule 1), SELE (E-selectin), SELP (P-selectin) and ITGβ2 (integrin β2) consistent with endothelial inflammatory activation and leukocyte recruitment during BBB dysfunction (101).

Systems-level analyses further support this integrated model of BBB disruption. High throughput quantitative proteomics of RA-infected duck brain tissues identified significantly enriched MAPK signaling pathway, Rap1 signaling pathway, motor proteins, endocytosis, regulation of actin cytoskeleton, focal adhesion, tight junction, necroptosis, apoptosis and gap junctions. Transcript-level validation further confirmed a highly coordinated upregulation of DOCK1, PYGL, Caspase-1, and A0A8B9ZV17 alongside the downregulation of PRKCZ, ADCY3, and protein A0A8B9QKV7 to drive endothelial cell migration, programmed cell death, TNF and NF-κB signaling, and endothelial cell permeability. Targeted Western blot analysis also confirmed downregulated expression of CDC42, a Rho GTPase protein family member essential for cytoskeletal support of endothelial cell adhesion, and the upregulation of Caveolin-2, an essential structural protein for the caveolae-mediated internalization, confirming that independent paracellular and transcellular pathways are actively hijacked by RA to drive meningitis (122). Collectively, these reports establish a comprehensive mechanistic framework supporting the complementary transcellular and paracellular pathways of RA neuroinvasion. Nevertheless, the host receptor for OmpA-mediated endothelial invasion in ducks remains unidentified, representing a critical knowledge gap in the molecular basis of BBB invasion.

### 3.6 Pathological manifestations and organ tropism

Gross pathological findings in affected ducks are primarily characterized by severe fibrinous polyserositis and multisystemic inflammatory lesions in the heart, liver, air sacs and respiratory tract, often accompanied by pneumonia, parenchymatous congestion, and swollen livers with multifocal necrotic foci (16, 48, 68). RA infection is histopathologically characterized by edema, hemorrhage, abundant fibrin deposition, heterophilic and macrophage infiltration, multinucleated giant cell formation and varying degrees of tissue necrosis (5, 68, 120). Cardiac lesions are characterized by pronounced fibrinous pericarditis, intermyofibrillar separation, cardiomyocyte degeneration, edema, hemorrhage and extensive heterophilic and macrophage infiltration (5, 16, 120). Hepatic lesions generally show hepatocellular enlargement, perivascular lymphocytic infiltration, congestion, fatty degeneration, steatosis, inflammation, and multifocal necrosis, whereas splenic pathology ranges from fibrinoid necrosis and characteristic “marble-like” necrosis to severe lymphoid depletion and disruption of red and white pulp architecture (5, 16, 35, 120, 129). Neuropathological lesions include meningitis, heterophilic and fibrinous ventriculitis, mild cerebral edema, neuronal hyperchromasia, and neurophagia, and lesions are frequently more severe in the brain stem and optic lobes than in the cerebrum, cerebellum, or spinal cord (5, 68, 120). Enlarged kidney and renal hemorrhage have also been reported, further supporting the multisystemic nature of RA-associated injury (32, 120). Interestingly, in some cases the spleen represented the only organ with overt lesions, appearing as characteristic white discolorations or “spotted spleen,” despite minimal pathological alteration in other major organs, suggesting selective targeting of RA on lymphoid and reticuloendothelial tissues involved in systemic immune regulation and bacterial clearance (35).

### 3.7 Host adaptation and expanding disease spectrum

Compared to ducks, marked host-associated differences in lesions, tissue tropism, and disease severity have been documented among geese, turkeys and chickens (**Table 1**). Although ducks and geese share the classical septicemic-serosal disease pattern, affected geese additionally showed synovitis of the tibio-tarsal joints and severe edema surrounding the metatarsus and tibio-tarsal region, with outbreaks characterized by neurological signs, 20-30% morbidity and 5-20% mortality (2, 37). Similarly, turkeys develop respiratory disease and fibrinous serositis, although disease is more common in birds between 6–15 weeks of age, with mortality ranging from 5-46% in commercial meat-type turkeys (67). In contrast, RA pathobiology in chickens appears more heterogenous and may reflect ongoing host adaptation. Although earlier reports suggest that chickens are relatively more resistant to RA and have lower mortality rates compared to ducks, recent investigations increasingly associate RA with airsacculits, serositis, pericarditis, perihepatitis, and edematous swelling in tibio-tarsal joints, and one investigation detected RA in 41.77% of chickens presenting with leg/joint abnormalities (5, 41, 50, 72). Comparative experimental infection studies have found host-associated differences in RA. Although chicken-derived serotype 1 strains showed increased adhesion (0.13%) and invasion (0.012%) capacities in primary chick embryo fibroblasts (CEF) compared to duck embryo fibroblast (DEF) along with an increased survival rate (47-121%) in chicken serum, experimental infection still resulted in substantially lower mortality in chickens (10%) than ducks (60%) and comparatively milder visceral pathology in chickens. The lesions in chickens are histopathologically characterized by localized hepatic congestion, sparse inflammatory infiltration, mild endocardial edema, and limited neuroinflammatory changes, whereas ducks developed fibrinous pericarditis, hepatocellular enlargement with perivascular lymphocytic infiltration, and pronounced neuropathological lesions. Bacterial burden analysis further demonstrated dynamic host-specific dissemination patterns in tissues, where chickens showed higher early bacterial loads in the liver and spleen, followed by decreased bacterial recovery at later stages of infection, while ducks maintained greater bacterial persistence during later stages of infection, particularly in the brain at 3–6 days post-infection (dpi) (5). Conversely, duck-derived serotype 1 strain showed higher adhesion (0.053%) and invasion (0.0047%) rates in DEF than in CEF, and higher bacterial burdens in the spleens and livers of infected ducks at 4-dpi, whereas there was greater persistence in both organs by 7-dpi in chickens (5, 72). Collectively, these findings highlight substantial host- and strain-associated differences in RA pathogenesis, demonstrating that variation in tissue tropism, bacterial persistence, lesion severity, and disease outcome likely reflects differences in host adaptation and immune responses among avian species. Although the molecular mechanisms governing these host-associated differences remain incompletely understood, emerging comparative studies, primarily in ducks and chickens, suggest that differential immune recognition, inflammatory regulation, and host-pathogen interactions likely contribute to the observed differences in disease susceptibility, tissue tropism, and pathogenesis among avian hosts. These comparative immunological mechanisms are discussed in greater detail in Section IV and are further summarized in **Table 4**.

**Table 4:** Comparative summary of host immune response against *R. anatipestifer*.

Stage of immunity	Species specific response	Defense mechanism	Key regulator/s	Functional immune response	Reference/s
Early Immune Response	Duck	PRR-associated signaling cascade	TLR 4	Ceca: receptor, results to downstream recruitment of MYD88 and TRAF-6	(130)
				Spleen and liver: receptor, results to ↑of pathological IL-17-associated response (IL-17A, IL-17F, IL-6, IL-8, IL-10, IL-1β, IL-22, and STAT3)	(72, 73, 131–136)
			TLR 3, TLR 4, TLR 7	Blood and brain: receptors, results to ↑ of inflammatory and immune-related transcripts (including IL-1β, IL-2, IL-4, IL-17A, IL-17D, IL-17F, and TGF-β)	(137)
		Complement and Coagulation Cascade	Complement proteins (C1R, C1S, C5, C9)	Activation of classical complement signaling, terminal complement effector pathways, and complement-coagulation crosstalk to mediate pathogen clearance and hemostasis regulation	(129)
			Coagulation-associated genes (F5, 13A1, PROCR)		
	Chicken	PRR-associated signaling cascade	TLR-3-STING-associated intracellular sensing axis	Liver, spleen, and cecal tonsils: receptor and regulator, sustained induction of IL-2, IL-8 and IFN-γ	(5)
			TLR 3, TLR 4, TLR 7	Tissue specific temporal activation resulting in coordinated immune regulation Liver: receptors, sustained activation Spleen: receptors, transient expression (TLR4 and TLR7)	(72)
			Inflammatory genes NOS2 and CCL5	Enhanced direct antimicrobial defense, and immune cell recruitment and signaling	(75)
Cell Mediated Immunity	Duck	T cell associated cytokine networks	Th17 associated cells	Splenic lymphocytes: coordinated ↑ of IL-17A, IL-17F, IL-6, TGF-β, IL-1β, IL-23p19, IL-12p40, IL-22 and γc	(72, 73, 133–135, 138)
				Spleen: ↑ IL-17A, IL-17F, IL-26, CCL15, CCL4, IL-8L1, and NOS2 as a coordinated activation of cytokine signaling	(129)
		Key cytokines	IL-17A	Proinflammatory cytokine that promoted progressive inflammatory responses and linked to immune disequilibrium and progressive tissue pathology	(72, 73, 129, 133–135, 139)
			IL-23p19	Dispensable for initiating early hyper-inflammatory IL-17 associated response but may sustain or reinforce ongoing inflammatory polarization in later stage of infection	(134)
			IL-26	Candidate upstream inflammatory regulator associated with IL-17-associated immune pathways	(129)
			IL-22	Early immune coordination and local tissue protection	(135)
	Chicken	T cell associated cytokine networks	Th1- and Th-2 associated cells	Spleen and liver: ↑ IL-2, IL-4, IL-8, IL-10 and IFN-γ. These cytokines are capable of preserving antibacterial defense while limiting excessive inflammatory injury	(5, 72, 73, 140)
		Other immunoregulatory networks		•↑ regulatory mediators (NOS2, IL-1β, CXCL14, TNIP3 and IL13RA2) •IL-17-associated signaling components (IL-17A, IL-17F, IL-6R, MAP3K7CL, and IRF4) exhibited time-dependent ↓, indicating coordinated immune regulation that restrains excessive inflammation while sustaining antimicrobial activity and chemotactic signaling	(75)
Mucosal and Humoral Immunity	Duck	Structural barrier defense	Epithelial transport associated genes and tight junction proteins	↓ AQP2, AQP3, AQP10, SGLT-1, PepT1, CLDN-1, OCLN, ZO-1: results to compromised epithelial integrity, increased permeability, and impaired absorptive function of the intestinal mucosa	(141)
			Mucin	Mucus-associated defense	(130)
		Humoral Immune response	secretory immunoglobulin A (sIgA)	The predominant antibody isotype that prevented bacterial adherence and epithelial invasion through immune exclusion, neutralizing virulence factors, and promoting pathogen clearance	(142–145)
			Maternal Ab or RA-specific IgY Ab	Early but transient passive protection	(146, 147)

↑, upregulated/increased expression; ↓, downregulated/reduced expression; γc, common receptor gamma chain; Ab, antibodies; AQP2, Aquaporin-2; AQP10, Aquaporin 10; CLDN, Claudin; CXCL14, C-X-C motif chemokine ligand 14; IFN-γ, Interferon-gamma; IL, interleukin; IRF4,; Interferon regulatory factor 4; NOS2, Nitric Oxide Synthase 2; MAP3K7CL, Mitogen-activated protein kinase kinase kinase 7 C-terminal like; OCLN, Occludin; PepT1, Peptide Transporter 1, SGLT1, Sodium/Glucose cotransporter 1; STAT3, Signal Transducer and Activator of Transcription 3; TGF-β, Transforming growth factor-beta; TLR, Toll-like receptors; TNIP3, TNFAIP3 interacting protein 3; ZO-1, Zona Occludens 1.

An additional and increasingly important aspect of RA pathobiology is its association with reproductive tract. Earlier reports have recognized the involvement of RA in waterfowls but its pathobiological consequences in commercial chickens have only recently become more recognized. In an extensive investigation of salpingitis in ducks and geese, 1,041 RA isolates were assigned to at least eight serotypes, predominantly serotypes 1, 9, 3 and 8, indicating that reproductive tract-associated infection was not restricted to a single serotype (43). However, the absence of genomic comparisons with conventional respiratory or septicemic isolates limits the determination of their genetic lineages and potential genotype-tissue tropism associations. Recent studies in chickens extend these observations, although differences in phylogenetic resolution preclude direct lineage-level comparisons. Multi-locus sequence typing (MLST) and pan-genome analysis identified the chicken-derived strain S63 as a novel sequence type occupying a distinct phylogenetic branch (148). By contrast, laying-hen isolate CK-RA-SD24 clustered with established duck-derived strains strains (RA serotypes 2 RAf55 and serotype 10 HXb2) based on selected-gene phylogenetic analyses (ompA, rpoB, prtC, hagA, and sspA) (40). Under their respective experimental conditions, both isolates caused reproductive impairment including declined egg production and hatchability (40, 148). Consistent with the broader pathological relevance of reproductive tract involvement, a cross-sectional investigation in chicken farms and hatcheries identified oviduct as the tissue with the highest RA isolation rate (21.68%), with RA also recovered from 20.18% of the examined dead embryos (14).

The transmission routes underlying reproductive involvement also still remain uncertain. Although experimental findings with S63 suggested possible semen-mediated transmission from roosters to hens and subsequent transmissions to hatchlings, alternative exposure routes were not systematically excluded (148). Moreover, the absence of RA isolation from semen and ovarian follicles in previous large-scale epidemiological survey leaves whether true venereal or vertical transmission occurs unresolved (14). Collectively, current evidence establishes reproductive disease as an increasingly recognized component of the expanding pathological spectrum of RA but does not establish distinct reproductive pathotype. Whether reproductive involvement reflects tissue-specific genetic adaptation or the broader capacity of circulating strains to colonize multiple tissues remains unresolved. Comparative genomic and functional analyses of reproductive tract-derived, respiratory, and septicemic isolates are therefore required to define the determinants of host adaptation, oviductal colonization, reproductive pathology and transmission.

Overall, current evidence suggests that RA pathobiology is driven by a coordinated interplay of virulence-associated secreted factors, iron acquisition pathways, immune evasion mechanisms, and host-adaptive traits that promote systemic dissemination, neuroinvasion and multisystemic inflammatory injury in avian hosts. Comparative pathological findings also reveal expanding pathological landscape of RA and the pronounced host-associated differences in tissue tropism, bacterial persistence, lesion severity, and disease outcomes, highlighting the complex nature of RA-host interactions. Despite recent advances in characterizing RA virulence determinants and pathological manifestations, the immunopathological mechanisms governing host susceptibility, bacterial clearance, neurotropism, and durable protective immunity are still poorly understood. Foremost, the host cell surface receptors mediating OmpA-dependent adhesion and invasion in duck cells remains currently unidentified. Determining whether RA OmpA engages avian orthologs of endothelial cell glycoprotein 96 (Ecgp96) and scavenger receptors LOX-1 and SREC-I, analogous to those exploited by *E. coli* K1 and *Klebsiella pneumoniae*, respectively, would substantially advance our understanding of the molecular mechanisms underlying OmpA-mediated transcellular invasion and identify potential targets for therapeutic and preventive strategies (149, 150).

## 4 Host immune responses and differential host immunodynamics during RA infection

The immunological characteristics of RA are characterized by a coordinated yet dynamic interplay between innate and adaptive immune pathways that collectively determine host susceptibility, bacterial clearance, disease severity, and recovery outcome. Following infection, RA is recognized by host pattern recognition receptors (PRRs), in particular Toll-like receptors (TLRs), which initiate early inflammatory signaling cascades and shape downstream adaptive immune polarization (72, 137). The activation of TLR-associated pathways, including myeloid differentiation factor 88 (MYD88)- and TNF receptor-associated factor 6 (TRAF6)-mediated signaling, promotes the production of cytokines, chemokines, and antimicrobial mediators that orchestrate leukocyte recruitment and antibacterial defense (130). These early innate sensing mechanisms subsequently drive distinct T helper (Th) cell differentiation pathways, which may vary among avian hosts, tissue compartments, and infection stages.

Cytokines are small soluble proteins that mediate communication between immune and non-immune cells and function as principal immunoregulatory mediators to regulate immune cell activation, proliferation, differentiation, and effector functions (151, 152). These molecules are mainly produced by macrophages and lymphocytes, although epithelial cells, endothelial cells, dendritic cells, mast cells and polymorphonuclear leukocytes (PMN), also contribute to cytokine production to coordinate innate and adaptive immunity and promote pathogen clearance and tissue protection (152–154). However, dysregulated cytokine production or signaling can contribute to pathological conditions associated with infectious diseases, cancer, chronic inflammatory disorders and autoimmune diseases (152, 153, 155–157).

**Figure 2:**
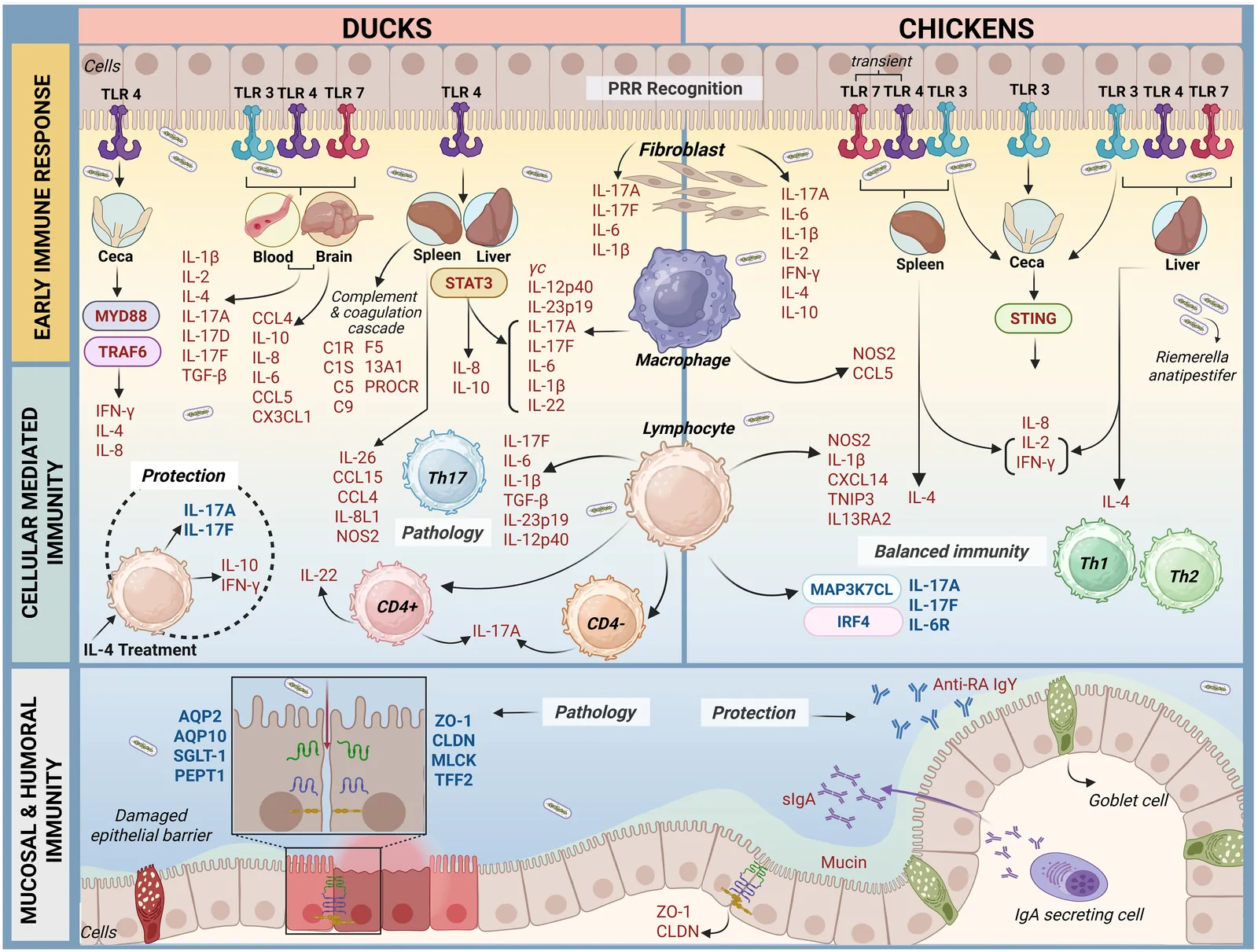
Host immunological landscape in *Riemerella anatipestifer* (RA) infection. Current evidence supports a coordinated, multi-layered immune response involving innate, cellular, mucosal, and humoral immunity in RA. RA is recognized by Toll-like receptors (TLRs), triggering inflammatory responses and complement and coagulation cascades. Comparative studies within macrophages, fibroblasts and tissues of RA infected ducks and chickens show that chickens preferentially activate a coordinated TLR 3 associated signaling whereas ducks exhibit stronger and more sustained TLR 4 signaling and inflammatory response (Upper panel). Antigen presentation activates a more balanced Th1-Th2 cytokine profile that supports effective bacterial clearance with limited immunopathology in chickens. In contrast, ducks display exaggerated pro-inflammatory responses characterized by sustained Interleukin-17A (IL-17A) and Th17 related cytokine profile expression. Both CD4+ and CD4- cells contribute to IL-17A production, while suppression of IL-17A/IL-17F alleviates immunopathology (Middle panel). RA infection also disrupts epithelial barrier integrity through reduced mucin production and downregulation of epithelial transport proteins (AQP2, AQP10, SGLT1, and PEPT1) and tight junction proteins (ZO-1, CLDN, MLCK, and TFF2) leading to pathology. Whereas, protective and mucosal immunity restores barrier function by enhanced secretory IgA (sIgA), RA-specific IgY, mucin secretion and tight junction protein expression (Lower panel). Red text indicates upregulated expression, whereas blue text indicates downregulated expression of genes, proteins, cytokines, or signaling pathways. Solid arrows indicate the presence and direction of reported signaling, cellular responses, or biological outcomes while brackets indicate receptors, cells, tissues, or immune mediators associated with the same response. The dashed outline groups IL-4 associated immune changes in ducks linked to protection and does not represent a discrete signaling pathway. AQP2, Aquaporin 2; AQP10, Aquaporin 10; CCL4, C-C motif chemokine ligand 4; CCL5, C-C motif chemokine ligand 5; CLDN, Claudin; CXCL14, CXC Chemokine Ligand 14; IFN-γ, Interferon gamma; MLCK, Myosin Light Chain Kinase; NOS2, Nitric Oxide synthase 2; PEPT1, Peptide Transporter 1; SGLT1, Sodium/Glucose cotransporter 1; TFF2, Trefoil Factor 2; TGF-β, Transforming Growth Factor beta; γc, Common Receptor Gamma Chain; ZO-1, Zona Occludens 1. The illustration is created using Biorender (https://BioRender.com/4ef36r0).

Recent advances in molecular and omics-based immunoprofiling approaches have substantially expanded the current understanding of the avian host response to RA infection, revealing previously unrecognized complexity in cytokine regulatory networks and immune signaling pathways. Emerging evidence suggests that RA-induced immunity extends beyond the classical Th1/Th2 paradigms and involves unique avian immunological features, including non-canonical Th17-associated signaling, tissue-specific immune regulation and highly divergent host immune trajectories. Accordingly, this section focuses on the immunological responses during RA infection to elucidate a mechanistic understanding of host-pathogen interactions and inform potential host-directed immunomodulation targets (**Figure 2**; **Table 4**).

### 4.1 Innate immune recognition and early host defense

Host systemic and mucosal recognition of RA entry initiates an innate immune response orchestrated through specialized PRR-associated signaling cascades, via TLR 3, TLR 4, and TLR 7, in target tissues including the blood, spleen, liver, brain, and cecal mucosa (72, 130, 137). Activation of these receptors subsequently engages downstream intracellular signaling cascades that coordinate early inflammatory responses through induction of cytokines, chemokines, nitric-oxide associated pathways, and antimicrobial effector molecules for bacterial containment (72, 73, 130, 137, 158). However, the magnitude and orientation of these innate responses diverge significantly between susceptible and resistant hosts. Substantial genetic diversity among duck populations has been previously reported, and recent comparative studies have further demonstrated breed-associated differences in cytokine regulation, immunoglobulin responses, and iron homeostasis following RA challenge (74, 159). Native Ji’an Red-feathered ducks showed greater resistance and more coordinated IL-17A-IgA-associated immune responses compared to commercial White Kaiya ducks (74). Together, these findings highlight the critical role of host genetic architecture in shaping susceptibility, immune regulation, and disease resilience in RA infection.

The mechanistic recognition of bacterial cell wall structural components of RA in the ceca of ducks strongly activates the TLR 4 signaling cascades and recruits downstream MYD88 and TRAF-6 and transcriptional upregulated expression of IFN-γ, IL-4, and IL-8 (130). Complementary analyses in RA-infected ducks have further shown that the activation of TLR 3, TLR 4, and TLR 7 occurs in the blood and brain during early infection and is accompanied by the increased expression of IL-1β, IL-2, IL-4, IL-17A, IL-17D, IL-17F, and TGF-β, which supports the rapid integration of innate inflammatory sensing with early immune polarization during systemic dissemination (137). Additionally, in the brains of infected ducks, the mRNA transcripts of chemokines and cytokines CCL4, CCL5, CX3CL1, IL-8, IL-6 and IL-10 were upregulated (101). In the spleens and livers of RA-infected ducks, substantial upregulation of TLR4 and the activation of downstream immunity to a pathological IL-17-associated inflammatory phenotype with marked upregulated expression of IL-17A, IL-17F, IL-6, IL-8, IL-10, IL-1β, IL-22, and STAT3 were consistently observed, which together supports the development of a hyper-inflammatory cytokine milieu associated with severe systemic pathology (72, 73, 131–136). Interestingly, although early RA infection was associated with increased IL-17D expression in blood and brain tissues, progressive infection in splenic and hepatic tissues showed reduced IL-17D together with the suppression of splenic TGF-β, suggesting temporal and tissue-specific remodeling of cytokine networks that favors a sustained pro-inflammatory state during severe infection (72, 131).

Complement-associated inflammatory signaling also appears to contribute significantly to RA-induced immunopathology. As discussed in the pathobiology section, RA has various complement evasion mechanisms to evade complement-mediated bacterial clearance. In the host, transcriptomics analysis of RA-infected spleens revealed significant enrichment of the complement and coagulation cascade pathway with the upregulation of complement components C1R, C1S, C5, and C9, as well as coagulation-associated genes such as F5, 13A1, and PROCR (129). These findings suggest that the host likely attempts to counteract RA with the activation of classical complement signaling, terminal complement effector pathways, and complement-coagulation crosstalk. However, complement activation appears to exert both protective and pathological effects. Functional studies have found that a blockade of the host C5a-C5aR axis in RA-infected ducklings significantly reduced mortality and bacterial burden in the peripheral blood and liver tissues, alleviated tissue damage in the heart, liver and spleen, and decreased the expression of inflammatory cytokines (160). Collectively, these observations suggest that RA pathogenesis involves bidirectional host-pathogen interactions characterized by simultaneous bacterial complement evasion and sustained host complement-mediated inflammatory amplification, where complement activation likely plays a role in host protection, but dysregulated downstream signaling also contributes to systemic pathology and disease progression.

Previous reports also suggest that RA-induced inflammatory amplification originates early in innate immune compartments before progressing to systemic adaptive Th17 polarization. *In vivo* analyses in the spleens and livers of RA-infected ducks showed significant upregulation of both membrane-associated common cytokine receptor γ chain (*γc*-a) and soluble receptor γ chain (*γc*-b), along with a marked increase in IL-17A and IL-17F, suggesting the coordinated activation of *γc*-associated signaling and IL-17 related inflammatory networks during acute infection. In the same study, RA stimulation of duck macrophage induced coordinated upregulation of *γc*, IL-17A, IL-17F, IL-6, and IL-1β, while functional silencing of *γc* using siRNA attenuated the expression of IL-17A, IL-17F, IL-6, and IL-1β, and simultaneously increased IFN-γ and IL-2, supporting the role of *γc* signaling in shaping inflammatory cytokine polarization during RA infection (133). Independent studies in RA-stimulated duck macrophages further demonstrated sustained upregulation of IL-17A, IL-17F, IL-22, IL-23p19, and IL12-p40, suggesting that macrophage activation may establish an inflammatory environment favorable for downstream Th17-associated immune response (134, 135).

Comparative *in vitro* analyses using duck embryonic fibroblast (DEF) and chick embryonic fibroblast (CEF) highlighted host-associate differences in early inflammatory coordination following RA-stimulation. Both DEF and CEF models showed upregulation of IL-17A, IL-6, and IL-1β following infection; however, DEF exhibited stronger and more sustained expression, including IL-17F, whereas CEF displayed comparatively balanced cytokine responses characterized by increased IL-2, IFN-γ, IL-4 and IL-10 expression (140). *In vivo* gene expression analysis of chicken and duck tissues revealed the divergent activation of innate sensing and cytokine pathways between species. Chickens preferentially activated a TLR-3-STING-associated intracellular sensing axis characterized by consistently higher TLR3 expression in the liver, spleen and cecal tonsils, elevated splenic and temporally increased STING expression, and sustained induction of IL-2, IL-8 and IFN-γ (5). Complementing these enhanced innate response, stimulation of HD11 macrophage cells with heat-killed RA upregulated the inflammatory genes NOS2 and CCL5, suggesting enhanced direct antimicrobial defense, and immune cell recruitment and signaling (75). Additional comparative analysis showed that RA-infected ducks have more sustained and consistent TLR4 expression in both spleen and liver at all time-points, while RA-infected chickens exhibit tissue-specific temporal TLR activation characterized by sustained hepatic TLR3 (1,4,7,10 dpi), TLR4 (1,4,7,10 dpi) and TLR7 (4,7,10 dpi) but only transient expression of TLR4, and TLR7 in the spleen (7dpi) (72). Collectively, these findings suggest that early host cellular responses may already predispose downstream trajectories to either inflammatory escalation in susceptible ducks or coordinated immune regulation in chickens.

Overall, current evidence supports a model in which RA infection initially activates host protection through PRR-mediated sensing and inflammatory containment. However, sustained activation of TLR4-associated pathways, *γc-*dependent cytokine amplification, and persistent IL-17-associated inflammatory signaling progressively shift innate responses from bacterial control to excessive inflammatory signaling and collateral tissue injury. Importantly, comparative studies of duck breeds and avian species indicate that the disease outcome is determined not only by the magnitude of immune activation, but by the host capacity to regulate inflammatory polarization and preserve immune homeostasis. These findings, taken together, suggest that early innate immune programming acts as a critical immunological checkpoint that shapes subsequent adaptive immune development and ultimately influences T-cell differentiation, cytokine polarization, and the balance between protective immunity and immunopathology during RA infection.

### 4.2 Cellular adaptive immunity and T-cell polarization

Following early innate inflammatory programming, RA infection progresses to adaptive immune activation characterized by dynamic cross-talk between inflammatory sensing pathways and coordinated T-cell polarization networks that collectively determine pathogen clearance, immune memory and disease outcome. Differentiation of naïve CD4 T-lymphocytes results in at least three major Th cell lineages, namely Th1, Th2 and Th17, each characterized by specialized cytokine profiles and immunological functions (161, 162). Among the major adaptive immune axes, Th1-associated cytokines including interferon-γ (IFN-γ), tumor necrosis factor-α (TNF-α), and interleukin-2 (IL-2), increase macrophage activation and bacterial clearance, whereas Th2-associated cytokines such as IL-4 and IL-10 contribute to humoral immune regulation and the maintenance of immune homeostasis (73, 151, 161). In contrast, Th17-associated cytokines, including IL-17A, IL-17F, IL-21 and IL-22, are closely associated with mucosal immunity, heterophil recruitment, epithelial barrier defense, and antimicrobial peptide induction (151, 161, 162). Current evidence indicates that RA infection induced substantial remodeling of these T-cell associated cytokine networks, characterized by differential activation of Th1-, Th2- and Th17-associated immune programs across host species, tissues, and disease severity, where excessive IL-17A-associated activation appears to be closely linked to progressive immunopathology in susceptible hosts.

Although Th17 differentiation pathways are well characterized in mammals, the molecular regulation of this lineage in avian species, particularly ducks, remains incompletely defined. Based on conserved vertebrate immune mechanisms, IL-6, TGF-β, IL-1β, and IL-23 are considered important regulators of Th17-associated responses with IL-6 and TGF-β primarily contributing to early lineage commitment and IL-23 supporting the expansion and stabilization of differentiated Th17 cells (73, 134, 135, 162). However, whether these cytokine requirements are identical in ducks remains unclear. During RA infection, coordinated induction of IL-6, TGF-β, IL-1β, IL-23p19, IL-12p40, IL-17A, IL-17F, and IL-22 has been observed in duck lymphoid tissues and stimulated splenic lymphocytes, suggesting activation of a conserved Th17-associated immune response (72, 73, 134, 135, 138). In addition, transcriptomic analysis identified RORA, a gene associated with mammalian Th17 differentiation pathways, among the upregulated genes in RA-infected duck spleens (129). While the RORγt transcription factor serves as the lineage-defining regulator of mammalian Th17 cells, the functional contribution of duck RORA/RORγ-related signaling during RA infection remains experimentally uncharacterized and is currently inferred from conserved immune pathways and comparative avian immunology (163).

Among susceptible avian hosts, adaptive responses during RA infection appears to preferentially polarize to a Th17-associated inflammatory phenotype. Multiple gene expression analysis studies using RA-stimulated duck splenic lymphocytes have demonstrated coordinated upregulation of IL-17A, IL-17F, IL-6, TGF-β, IL-1β, IL-23p19, IL-12p40, IL-22 and *γc*, supporting the rapid activation of inflammatory T-cell programs following antigenic stimulation (72, 73, 133–135, 138). Additional gene expression profiling in the spleens of RA-infected ducks showed highly regulated immune-associated transcripts including IL-17A, IL-17F, IL-26, CCL15, CCL4, IL-8L1, and NOS2, suggesting coordinated activation of inflammatory cytokine signaling, chemokine-mediated immune recruitment, and antimicrobial effector responses. In the same study, pathway enrichment analysis using the Kyoto Encyclopedia of Genes and Genomes (KEGG) identified significant enrichment of the Th17 cell differentiation pathway, supporting the role of IL-17 mediated immunity in RA infection (129).

However, temporal comparisons suggest that adaptive cytokine polarization during RA infection may not follow fully canonical Th17 differentiation pathways. Although IL-23p19 was induced in stimulated splenic lymphocytes and became elevated during later infection stages in the spleen of RA-infected ducks, minimal changes in IL-23p19 expression were detected during early *in vivo* infection, despite substantial induction of IL-17-associated responses (134). These findings suggest that the initiation of adaptive inflammatory responses during RA infection may occur independently of early IL-23 signaling, whereas IL-23 may function later to sustain or reinforce inflammatory polarization rather than initiate it. Recent transcriptomics analyses showed concurrent induction of IL-26 and IL-17A-associated cytokines and identified IL-26 as a candidate upstream inflammatory regulator associated with IL-17-associated immune pathways (129). Given its predicted interaction with inflammatory cytokine networks, IL-26 has been proposed to function as a molecular intermediary linking early inflammatory activation with sustained adaptive immune amplification, although direct mechanistic validation during RA infection remains to be further elucidated.

Adaptive cytokine responses during RA infection additionally demonstrate marked cellular specialization. Functional analyses revealed that IL-22 was predominantly secreted by CD4^+^ splenic lymphocytes, whereas IL-17A was induced on both CD4^+^ and CD4^-^ splenic lymphocyte populations, indicating broader deployment of inflammatory signaling beyond classical helper T-cell compartments. Consistent with these cellular findings, the *in vivo* expression kinetics of IL-22 and IL-17A revealed distinct tissue-specific patterns, with IL-22 showing rapid but transient induction and peaking in the spleen at 1dpi and in the liver at 4dpi before returning to baseline, whereas IL-17A showed stronger and more sustained induction (135). Together, these findings suggest that IL-22 functions primarily in early immune coordination and local tissue protection, while IL-17A signaling appears to promote progressive inflammatory responses in multiple cell populations during RA infection.

Interestingly, experimental RA-infection in murine models supports that excessive IL-17A-associated inflammation impairs host resistance. Despite relative intrinsic resistance to RA infection, administration of recombinant IL-17A (5µg) or IL-23 (5µg) prior to sublethal challenge significantly increased splenic bacterial burden, aggravated splenic enlargement, and increased disease severity in mice (139). These findings support a model in which sustained activation of pro-inflammatory cytokine, IL-17A signaling, redirect host protective immunity to immunopathology and impaired bacterial control. Also, the contribution of upstream IL-23 signaling may differ according to host species and infection stage.

In contrast to susceptible ducks, comparative host studies have shown that chickens preferentially activate coordinated Th1- and Th-2 associated immune programs characterized by increased expression of IL-2, IL-4 and IFN-γ in livers and spleens of RA-infected chickens, which collectively correlate with lower mortality following RA challenge (72). Mechanistically, the immunoregulation role of Th2-associated signaling was further demonstrated in RA-stimulated duck splenic lymphocytes, where recombinant duck IL-4 significantly suppressed expression of IL-17A and IL-17F while simultaneously increasing expression of IFN-γ and IL-10 (73). These findings suggest that adaptive counter-regulatory pathways may function as critical immunological checkpoints capable of restraining excessive inflammatory amplification and restoring immune equilibrium during RA infection.

Beyond Th2-associated regulation, anti-inflammatory cytokine networks may also contribute to the modulation of host responses during RA infection. Increased expression of regulatory cytokines, including IL-10 and TGF-β, has been observed during RA infection or following RA antigen stimulation, suggesting activation of immune regulatory mechanisms that may help limit excessive inflammatory responses (73, 134). In duck embryonic fibroblasts (DEFs), IL-10 expression was upregulated together with pro-inflammatory markers at later stages of RA stimulation, suggesting a potential feedback mechanism that may temper excessive inflammatory activation. In contrast, chicken embryonic fibroblasts (CEFs) exhibited relatively stable IL-10 expression during RA stimulation, indicating host-specific differences in regulatory cytokine responses (140). Similarly, TGF-β represents an important regulator of immune homeostasis in both ducks and chickens and has been implicated as a cytokine involved in Th17-associated differentiation pathways in avian species (73, 162). However, whether RA-induced IL-10 and TGF-β responses directly promote regulatory T-cell development or represent broader host mechanisms for controlling inflammation remains unclear.

Additional regulatory complexity appears to extend beyond cytokine transcription alone. Evidence from RA infection studies suggests that downstream inflammatory signaling pathways undergo dynamic regulation rather than simple activation. Gene expression and small RNA analyses of RA-stimulated chicken splenocytes elucidated coordinated yet temporally regulated remodeling of inflammatory and immunoregulatory networks characterized by induction of NOS2, IL-1β, CXCL14, bactericidal increasing protein-like genes, and regulatory mediators including TNIP3 and IL13RA2 at multiple timepoints. Interestingly, several IL-17-associated signaling components including IL-17A, IL-17F, IL-6R, MAP3K7CL, and IRF4 exhibited time-dependent downregulation, suggesting that chicken hosts may actively restrain excessive inflammatory amplification while maintaining antimicrobial and chemotactic defense responses. Conversely, sustained activation of IL-17-associated inflammatory pathways appears to be associated with increased immunopathology in susceptible hosts. Regulatory associations between gga-miR-465-3p-NOS2 and gga-miR-16-5p-CCL5 additionally support the role of post-transcriptional immune modulation in shaping a coordinated adaptive response during RA infection (75). Moreover, protein–protein interaction (PPI) network analysis of differentially expressed genes identified IL-17A as a central interacting node linked with IL-10, IL-26, NFKBIA, NFKB1, IL1R1, and IL2RA, highlighting the integration of IL-17-associated responses with NF-κB-mediated inflammatory regulation, cytokine signaling, and immune regulatory pathways (129). Together, these observations support a model in which post-transcriptional immune regulation contributes to inflammatory-regulatory cross-talk during RA infection and therefore presents a potential target for future immunomodulatory strategies. However, the extent to which these regulatory networks directly influence host resistance, tissue pathology, or therapeutic responsiveness remains incompletely resolved and therefore requires functional *in vivo* validation to establish causal roles in disease mitigation and host protection.

Together, these findings establish differential adaptive immune polarization and inflammatory-regulatory cross-talk as central determinants of host susceptibility and disease progression during RA infection. Susceptible hosts preferentially develop sustained IL-17A-associated inflammatory amplification linked to immune disequilibrium and progressive tissue pathology, whereas resistant hosts maintain more Th1-Th2-associated immune regulation capable of preserving antibacterial defense while limiting excessive inflammatory injury. Emerging transcriptional and post-transcriptional evidence further indicates that host-specific T-cell polarization is shaped through multilayered regulatory networks that integrate cytokine signaling, antimicrobial responses, chemotactic recruitment and inflammatory control.

### 4.3 Mucosal, humoral and passive immunity

Because RA commonly invades through skin abrasions, damaged epithelial surfaces, respiratory and gastrointestinal tract, mucosal and epithelial barriers constitute a major immunological interface central to early infection outcomes. Recent reports found that RA infection disrupts multiple mucus-associated and epithelial junction pathways that collectively compromise barrier integrity and facilitate progressive exudative pathology (27, 101, 122, 130). Consequently, an effective host defense relies not only on systemic inflammatory regulation and adaptive cellular polarization but also on the preservation of epithelial barrier integrity and a coordinated mucosal immune response capable of limiting bacterial adherence, colonization, and subsequent systemic dissemination.

Experimental infection studies have found that RA induces profound remodeling of the intestinal barrier, characterized by reduced mucosal thickness, shortened villi, decreased villus-to-crypt ratios, goblet cell depletion, and increased epithelial apoptosis (130, 141). These structural alterations are accompanied by significantly reduced expression of epithelial transport-associated genes and tight-junction proteins in the duodenum (Aquaporin 2/AQP2, Sodium/Glucose cotransporter 1/SGLT1, Peptide Transporter 1/PepT1), jejunum (AQP2, AQP3, AQP10, SGLT, Claudin-1/CLDN-1, OCLN, zona occludens-1/ZO-1), colon (AQP2, AQP3, AQP10, SGLT, PepT1, CLDN-1, OCLN, ZO-1), cecum (AQP2, AQP10, SGLT1, PepT1, CLDN-1, OCLN, ZO-1), and ileum (AQP2, SGLT1, PepT1) of RA-infected ducks, suggesting compromised epithelial integrity, increased permeability, and impaired absorptive function (141). In the same study, Alcian Blue and Periodic Acid-Schiff (AB-PAS)-stained cells were also markedly reduced in the colon of RA-infected ducks, indicating impairment of goblet cells and the protective mucus layer. RA infection induces temporal regulation of barrier reinforcement and tissue repair-associated genes and shows a continuous host compensatory response to pathogen-induced epithelial injury. In the ceca of infected ducks, early increases in ZO-1 and CLDN1 suggests the activation of mechanisms aimed at preserving epithelial integrity, whereas subsequent reductions in TFF2 (trefoil factor 2), ZO-1, CLDN2, and MLCK (myosin light chain kinase) indicate progressive impairment of epithelial recovery and mucosal repair. In contrast, MUC2 displayed a biphasic expression characterized by initial suppression followed by marked upregulation during the later stages of infection, consistent with compensatory activation of mucus-associated defenses (130). Transcriptomic analyses of RA-infected spleens also identified MUC2 and CLDN1 among the highly upregulated genes, although their biological significance in splenic tissue remains unclear (129). Taken together, current evidence suggests that RA drives progressive mucosal barrier dysfunction while simultaneously eliciting compensatory host responses directed toward epithelial repair, mucus layer reinforcement, and restoration of tissue homeostasis.

Beyond structural barrier defenses, humoral immune responses at the mucosal and systemic levels during RA infection have also been reported. Among the immune effectors operating at the mucosal surfaces, secretory immunoglobulin A (sIgA) is the predominant antibody isotype and constitutes a critical first line of defense through immune exclusion, blocking bacterial adherence and epithelial invasion, neutralizing virulence-associated factors, and facilitating pathogen clearance while minimizing excessive pathogen-mediated proinflammatory responses that could compromise mucosal integrity (142–145). Although the majority of IgA-producing cells are present in the lamina propria of intestinal mucosa, they are also widely distributed across multiple mucosal surfaces of the respiratory and genitourinary tracts, where they play pivotal role in protective immunity, pathogen clearance and homeostasis (143, 164–166). Evidence from experimental RA infection and immunization studies supports the involvement of both the systemic and mucosal antibody response in host defense. RA challenge studies in turkeys have demonstrated the development of RA-specific antibodies following aerosol, abdominal air sac injection and intratracheal inoculation, indicating robust activation of systemic humoral immunity (76). Complementing these findings, RA-specific sIgA was detected in the trachea, lungs and duodenum of ducks, and was significantly increased following immunization, showing the capacity of RA antigens to stimulate localized mucosal antibody responses at major sites of pathogen entry (167, 168).

Additional evidence linking humoral immunity to disease outcome was elucidated in a recent comparative infection study in resistant and susceptible duck breeds. Ji’an Red-feathered ducks were less susceptible to RA than White Kaiya ducks in the same infection conditions, indicating breed-associated differences. Between resistant and susceptible ducks in each breed, RA-susceptible Ji’an Red-feathered ducks showed significantly reduced IgA and stable IgG and IgM, whereas RA-resistant Ji’an Red-feathered ducks-maintained antibody profiles comparable to healthy controls. On the other hand, both RA-susceptible and RA-resistant White Kaiya ducks showed stable IgA compared to control, but RA-susceptible ducks increased both IgG and IgM responses while RA-resistant ducks displayed a moderate increase in IgM. Interestingly, susceptible White Kaiya ducks showed a marked reduction in IL-17A levels compared with resistant and control White Kaiya ducks. Furthermore, correlation analyses from the same study identified a positive correlation of IgA with IL-17A and IgG to IL-6, suggesting coordinated interactions between the humoral and cytokine-mediated immune pathways during RA infection (74). However, the precise contribution of each individual immunoglobulin isotypes and their relationship with Th17-associated immunity in mediating RA protection is still incompletely defined.

Maternal antibodies are pathogen-specific antibodies and represent an additional component of humoral immunity that is passed down from the hen to the chick through the egg yolk to provide the bird with early protection from diseases (146). Experimental studies have shown that RA-specific IgY antibodies induced in vaccinated parental ducks were efficiently transferred and provided passive protection, although levels declined between 3 and 10 days post hatch, indicating that the protection was transient and may explain the increased vulnerability of young ducklings as passive immunity wanes (147). While these findings show the potential importance of maternal antibodies in early host response, their duration, breadth of protection and contribution to disease resistance under field conditions are still unknown.

Current evidence supports epithelial barrier integrity and coordinated mucosal-humoral immunity as critical determinants of host defense against RA infection. While RA compromises the host’s first line of defense by disrupting goblet cell function, mucus production and tight-junction architecture, the concurrent host compensatory regulation of MUC2 and barrier-associated genes highlight an active attempt to preserve mucosal homeostasis. Additionally, protective immunity extends beyond localized barrier responses and involves integrated interactions between mucosal antibodies, systemic humoral immunity, and cytokine-mediated immune regulation. In particular, the studies on breed-dependent differences in antibody and cytokine profiles indicates that effective host resistance depends less on the magnitude of antibody production alone and more on the maintenance of coordinated mucosal, humoral, and cellular immune networks. However, current understanding of mucosal, humoral, and passive immunity against RA is derived predominantly from studies in ducks, whereas, comparable evidence in chickens remains scarce, limiting the generalization of these immune mechanisms and correlates of protection across avian species.

Taken together, immunity against RA is governed by a highly interconnected immunological network spanning innate recognition, adaptive immune polarization, mucosal barrier defense, and humoral immunity. Disease resistance appears to be associated with the preservation of mucosal barrier integrity, maintenance of balanced Th17-associated immune responses, induction of antigen-specific mucosal antibodies, and effective regulation of inflammatory amplification to prevent excessive inflammatory tissue injury. Consequently, future control and prevention strategies could benefit from approaches capable of simultaneous blocking of pathogen colonization at mucosal surfaces, supporting epithelial barrier homeostasis, and inducing appropriately regulated cellular immune responses capable of providing durable and potentially cross-serotype protection.

## 5 Vaccine development strategies for *Riemerella anatipestifer*

Over the years, antibiotics have traditionally been the standard control measure for riemerellosis. However, RA is currently resistant to antibiotics and many treatments have now become ineffective due to the emergence of multidrug-resistant strains (46, 169–171). Recently, the identification of RA carrying resistant genes such as *tetB*(P), *tet*(M), *tet*(T), *tet*(41), *tetA*(58), *tet*(O), *tet*(35) *tet(X)* and *tet(Q)* (tetracycline), *dfrA49* (trimethoprim), *OXA-209*, *erm*E, *erm*X, *ermF*, *floR*, and *ereD* has been one of the challenges to the prevention and control of the disease (16, 77, 171, 172). As a result, vaccination is currently considered the most effective long-term strategy for preventing RA outbreaks (36, 173). However, unlike many bacterial pathogens where limited serotypes dominate disease epidemiology, RA has various serotypes and varying regional distribution with little to no significant cross-protection among and between serotypes (167). Therefore, it is necessary to develop vaccines unique to the particular serotype circulating in a certain area (36, 174). This fundamental limitation in RA has transformed RA vaccine research from a simple-disease control measure to a continued effort to identify conserved protective antigens capable of eliciting broadly protective immunity across serotypes. **Supplementary Table 1** summarizes conventional, recombinant/subunit genetically engineered, and next-generation vaccines against RA to date.

### 5.1 Inactivated vaccines

Vaccine development for RA has evolved from conventional whole-cell bacterins to rationally engineered platforms designed to overcome the limitations of conventional vaccines. Inactivated vaccines (bacterins) are still the most widely used and commercially established strategy due to their inherent safety, scalability and capacity to induce high levels of homologous protection. Local strains such as serotype 1 broth culture bacterin (strain 1081) have demonstrated substantial protective indices (PI = 81.8-95.8) when administered in multi-dose by subcutaneous (SC) regimens (175). Early Montanide ISA 206-adjuvanted serotypes 1 and 2 also provided high levels of protection against homologous challenge (PI = 70-100) but poor heterologous protection (PI = 20-25), underscoring the limited cross-serotype immunity of conventional bacterins (176). Subsequent adjuvant optimization showed that levamisole-supplemented adjuvanted-serotype 2 vaccine provided complete homologous protection (PI = 100) and elicited sustained IgG responses with increased expression of IFN-γ, IL-2, IL-4 and IL-10 (177). Similarly, propolis-, oil emulsion-, and alum-adjuvanted serotype 1 bacterin formulations showed PI values of 83-92% (36). These studies indicate that while an adjuvant can substantially improve the magnitude of durability of bacterin-induced immunity and homologous protection, antigenic diversity remains the principal obstacle to achieve broad cross-serotype protection.

To overcome the intrinsic serotype restriction of monovalent bacterins, multivalent formulations such as bivalent vaccines targeting prevalent serotypes 1 and 2, and a highly protective trivalent-oil emulsion vaccines combining serotypes 1, 2, and 10, were developed to expand antigenic coverage against co-circulating field strains. These formulations achieved sustained antibody responses and protective indices of 85-100% against vaccine-matched serotypes while augmenting Th1- Th2- and antigen presentation-associated pathways, highlighting that broad antigenic coverage is an effective strategy to improve protection. (36, 174, 178). An extension of this multivalent antigen strategy has also been reported with the incorporation of RA antigens in combination with multiple economically important poultry pathogens. Formulations combining RA serotypes with *Escherichia coli* (*E. coli*), *Pasteurella multocida* (*P. multocida*), avian influenza virus (AIV) or duck hepatitis virus (DHV) achieved PI of 72.5-100% and maintained durable antibody responses against all vaccine components (179–182). Collectively, these findings demonstrate that broadening vaccine composition with the incorporation of multiple RA serotypes and co-circulating pathogens can achieve robust protection and simplify flock-level disease management. However, because protection is largely confined to represented serotypes, multivalent bacterins provide more of an epidemiological solution than a biological solution to antigenic diversity and necessitate continual reformulation as circulating serotypes evolve.

Beyond increasing antigen breadth with multivalent formulations, genetic engineering has also been applied to improve the cross-protective potential of conventional bacterins. Inactivation of transposon-derived mutants with disrupted lipopolysaccharide and O-antigen biosynthesis pathways generated antigen-modified vaccines capable of achieving PI values of 87.5-100% and 75-100% against homologous and heterologous challenge, respectively (183–186). These suggest that modification of immunodominant surface glycans can partially overcome serotype-restricted immunity by redirecting immune recognition to more conserved protective determinants. Therefore, inactivated engineered bacterins represent a vaccine platform that bridges the traditional whole-cell bacterins and modern antigen-directed vaccine strategies using targeted genetic modification to enhance cross-protective immunity while retaining the safety profile of inactivated vaccines.

### 5.2 Live attenuated vaccines

Live attenuated vaccines were developed to overcome the limitations of inactivated vaccines in enhancing breadth and quality of immunity by mimicking natural infection and stimulating an integrated mucosal, humoral and cellular immune response. Early approaches relied on either virulence-screened field isolates or empirical attenuation through serial laboratory passage. Oral vaccination with low-virulence serotype 1 (D15-RDA-92) or serotype 2 (D14-RDA-92) isolates in skim milk, either alone or as a bivalent formulation, induced mucosal sIgA responses and provided substantial homologous protection (PI = 60-100%), as well as broadened coverage and protection with the bivalent formulation against their respective serotype (PI = 70-100%) (167). Likewise, serially passaged serotype 2 attenuated strain elicited high antibody responses while generating persistent lymphocyte proliferative responses indicative of durable cell-mediated immunity (187). Both studies support the capacity of live vaccines to induce long-lasting systemic and mucosal immune memory. 

Although live attenuated vaccines have demonstrated promising protective efficacy against RA, the reliance on incompletely defined attenuation mechanisms raises concerns regarding residual virulence, genetic instability to revert to original virulence, environmental shedding, and inconsistent protection against heterologous serotypes, which warrants careful consideration, especially in actual production. Given the natural competence of RA and uptake of exogenous chromosomal DNA, it can facilitate intraspecies genetic exchange, resulting in genetic recombination and potential horizontal gene transfer (205). This characteristic of natural competence of RA, is associated with the *DprA* protein, which is vital for natural transformation, as well as other genes that are genetically encoded and regulated in RA such as *ComEC, ComF*, and *RecA* (94, 206). Hence, live attenuated vaccines require rigorous assessment regarding their safety, genetic stability and post-vaccination monitoring which are necessary prior to widespread application.

Advances in molecular genetics have subsequently transformed RA vaccine development from empirical attenuation to targeted disruption of defined virulence determinants. Metabolic, regulatory and transport mutants targeting transcriptional regulators (ΔB739_2187 mutant, also named K10 strain), nicotinamidase (Δ*pncA* mutant), iron acquisition (ΔB739_1343 mutant and Δ*ybiT*), ferric uptake regulator (Δ*fur* mutant), or two-component system regulator (Δ*phoP* mutant) consistently produced highly attenuated strains that retained strong immunogenicity, reduced bacterial colonization in tissues, and elicited elevated IgY responses and protective indices of 80-100% against homologous challenge (91, 188–192). While the Δ*fur* mutant combined rapid clearance and safety profile, it provided minimal heterologous protection (PI = 0-11.11%), highlighting the continued challenge of serotype-restricted immunity (191). Interestingly, deletion of the CRISPR-associated regulator *cas9* (Δ*Cas9* mutant) alone resulted in variable protection that was highly dependent on vaccination route and dose. Intranasal (IN) administration of Δ*Cas9* mutant at 3 x 10^5^ CFU/bird provided 80% homologous protection compared with only 30% following subcutaneous immunization, while it was 0-20% PI when given at lower dose (6 x 10^4^ CFU/bird) on either route (107).

Beyond metabolic and regulatory mutants, rational attenuation has also targeted structural and secretion-associated virulence determinants. Disruption of LPS-associated antigenicity through RAΔ604 mutant conferred 87.5% homologous protection following a prime-booster regimen, while deletion of the T9SS component *sprT* (*ΔsprT*) generated a highly attenuated strain capable of eliciting dose-dependent protection ranging from 60-100% from a single immunization (95, 193). More recently, a markerless Δ*cas9*Δ*sprA* double deletion mutant combining CRISPR-associated regulation and T9SS-linked iron acquisition defects induced robust systemic and mucosal immunity characterized by elevated serum IgY, IFN-γ, IL-6 and respiratory secretory IgA responses with IN and IM immunization conferring 100% homologous protection against similar strains and 70-90% PI against different strains (194). Collectively, these studies demonstrated that rational attenuation targeting multiple virulence pathways achieved high protective efficacy while retaining robust systemic immunity. The induction of respiratory sIgA and high protective index against RA infection by intranasally administered mutants (i.e., Δ*Cas9* and Δ*cas9*Δ*sprA*) further suggest that mucosal immunization may be a promising strategy for improving protective breadth beyond conventional live vaccine platforms. However, the extent of cross-protection across the broader diversity of RA serotypes and stability under commercial production conditions are still unknown.

Additionally, several genetically modified RA mutants targeting capsule biosynthesis, outer membrane, transport, and virulence-associated proteins (i.e., Δ*wza*, Δ*sprA*, Δ*gldK*, Δ*gldM*, Δ*gldG*, Δ*sspA*, Δ*OmpA*, Δ*OMP76*, Δ*OMP85*, Δ*OmpH* or ΔB739_0832) have shown varying degrees of attenuation, characterized by increased LD_50_ and reductions in one or more parameters associated with pathogenicity, including bacterial adherence and invasion, mortality, pathological lesions and bacterial colonization in blood and tissues relative to their wild-type counterparts (25, 26, 28, 80, 83, 97, 99, 100, 206, 207). Together, these findings highlight the contributions of these proteins to RA virulence and underscore their potential as vaccine targets, and several conserved candidates show promise for the development of broad-spectrum vaccines. However, their capacity to induce protective immunity and confer protection following vaccination-challenge studies warrants further investigation.

### 5.3 Recombinant subunit vaccines

Recombinant subunit vaccines were developed to overcome the biosafety concerns associated with live vaccines while enabling focused targeting of conserved RA antigens. Among the candidates evaluated, OmpA has emerged as the most extensively investigated antigen because of its surface exposure, conservation, and role in host-pathogen interactions. The formulation of a recombinant OmpA with CpG oligodeoxynucleotide adjuvant elicited sustained antibody responses detectable until 9 months post vaccination, increased expression of IFN-α, INF-γ, IL-6 and IL-12, and reduced pathological lesions following challenge, and generated serological reactivity against multiple RA serotypes (195, 196). Additionally, the fusion of OmpA to duck IgY Fc combined with *Schisandra chinensis* polysaccharide improved CD4^+^ and CD8^+^ T-cell responses and resulted in 86.7% homologous protection (197). However, not all conserved structural antigens are equally protective. Adjuvanted recombinant GroEL provided only partial protection (PI = 37.5-50), whereas GST-fused OmpA and P45N’ generated antigen-specific antibody responses but only conferred minimal protection (PI = 14.3), indicating that antigen immunogenicity does not completely translate into protective efficacy (198, 199).

Immunoproteomic approaches have also been employed to identify naturally immunodominant antigens recognized during infection. Recombinant cross-reactive proteins (rCR1, rCR4, rCR5) identified by immunoproteomic screening elicited measurable protection, with a combined antigen formulation outperforming individual recombinant protein vaccine counterparts ((PI = 50-62.5 vs. 0-60) (176). Similarly, targeting secreted virulence effectors has emerged as an alternative strategy to disrupt key pathogen mechanisms. Vaccination with recombinant T9SS-associated secreted protein PaR1 induced antigen-specific immunity but provided only modest protection (PI = 40) and failed to provide protection against heterologous serotypes (PI = 0-20) (200). Recombinant PorV, a T9SS component and adhesin, also induced specific antibodies with complement-dependent bactericidal and opsonophagocytic activities but conferred serotype dependent protection (PI = 40-75) (201). Taken together, these findings indicate that neither immunoreactivity nor functional antibody alone reliably predicts broad protection, underlining the need to evaluate antigen conservation, functional relevance and protective efficacy across diverse RA serotypes.

The identification of antigens expressed under host-relevant conditions presents another promising direction in vaccine development. A previous report on the immunization of proteins discovered in iron-restricted environments (i.e., Riean_1750 and Riean_1752) showed complete protection (PI = 100%) against homologous challenge when administered as a combined formulation following a primer-booster regimen (202). These studies support the concept that antigens associated with bacterial survival pathways may represent more effective vaccine targets than constitutively express structural proteins alone. Nevertheless, the excellent safety profile and manufacturing flexibility of recombinant subunit vaccines, most candidates continue to show variable protective efficacy and limited validation against genetically and antigenically diverse RA populations.

On the other hand, another relevant and critical issue in RA vaccine development and application that needs to be considered is the presence of maternally derived antibodies (MDAs) in ducklings from immune breeder flocks. Ducklings acquire RA maternal antibodies through egg yolk, which provides early passive protection during the first week of life (147, 208). However, passive protection rapidly declines within 5–10 days, and antibodies are serotype-specific (208). These circulating maternal antibodies may interfere with vaccination; hence, vaccination timing is important. Studies on the evaluation of MDA interference have been demonstrated in other avian vaccines but have not yet been established with RA vaccination. Nevertheless, this is a potential concern that needs to be considered, and further studies are needed to evaluate the impact of MDAs on the immunogenicity and protective efficacy of different RA vaccine platforms under field conditions.

### 5.4 Next-generation vaccines

Recent advances in RA vaccine development have increasingly focused on overcoming the limitations of conventional vaccine platforms with next-generation antigen design and delivery technologies. Emerging DNA-subunit prime-boost strategies have demonstrated the potential to enhance both the breadth and durability of immunity. Using the conserved OmpA antigen, an OmpA DNA-prime/protein-boost strategy (100ug OmpA DNA/rOMPA + 100ug CpG ODN) enhanced both the magnitude and durability of immune responses, extending peak antibody responses to 7–12 weeks, increasing CD8^+^ T-cell frequencies, upregulating expressions of IFN-, IL-6 and IL-12 and generating antibodies that recognize RA serotypes 1, 2 and 6 (209). However, whether these enhanced immunological responses translate into protection against virulence challenge is still unclear. Complementing these immune-engineering approaches are reverse vaccinology and in silico antigen discovery of conserved vaccine targets. A previous report on the highly conserved outer membrane assembly protein YaeT (OMP85 family) exemplifies this approach. In silico analyses of sequence homology, physiochemical and structural properties, transmembrane domains, and B-cell epitopes identified YaeT as a promising vaccine candidate. Subsequent immunization of recombinant YaeT (0.2mg/duck in Freud’s adjuvant given as primer-booster) elicited functional antibodies with complement-activating and opsonophagocytic activity and conferred 80% protection following RA serotype 1 challenge (203). Together, these studies highlight the utility of computation antigen discovery to accelerate rational RA vaccine design.

In parallel, advances in nanotechnology have enabled the development of targeted mucosal delivery systems designed to strengthen immunity at the primary portals of RA entry. Intranasal administration of calcium phosphate nanoparticle-conjugated outer membrane vesicles (CAP-OMV) induced robust systemic IgY and mucosal sIgA responses while achieving complete protection (PI = 100%) without detectable gross or biochemical toxicity (168). By combining the intrinsic antigenic complexity of OMVs with the delivery efficiency of biocompatible nanoparticles, this platform addresses the limitations of other traditional vaccine platforms in inducing protective mucosal immunity. More recently, *Salmonella* typhimurium-derived OMVs engineered to display RA antigens (PorQ, YaeTm or YiaD) elicited humoral and cellular immune responses and conferred 70-80% survival in vaccinated-infected ducks (204). These next-generation approaches indicate that future RA vaccine development may depend not only on identifying conserved protective antigens but also on optimizing antigen presentation, immune programming, and mucosal delivery. Nevertheless, most of these platforms are still at the experimental stage, and their protective breadth across the extensive serotype diversity in RA, long-term durability, scalability, and field applicability require further validation before commercial implementation.

**Figure 3:**
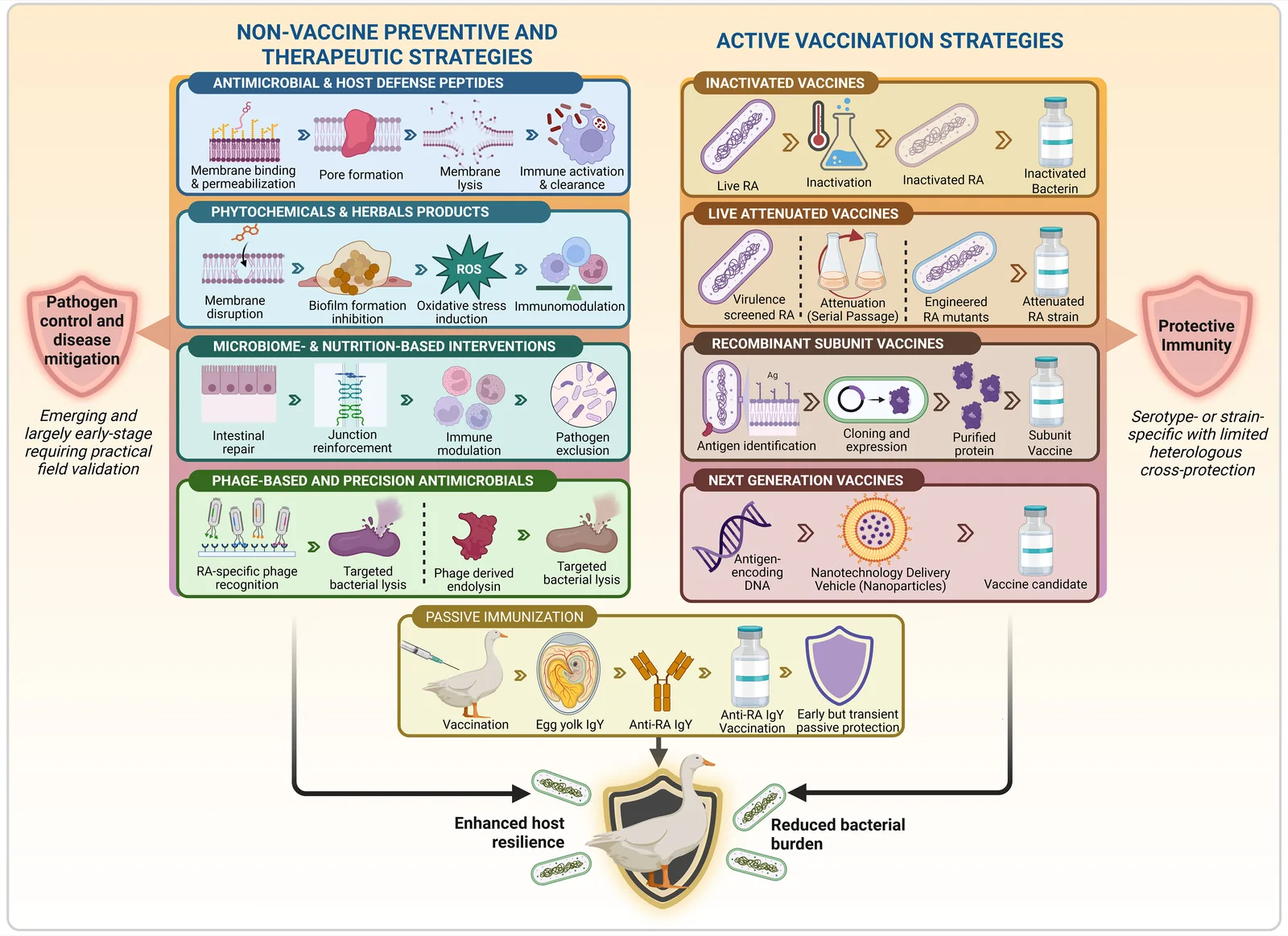
Current and emerging defensive strategies for *Riemerella anatipestifer* infection. The figure summarizes the major approaches developed to control *Riemerella anatipestifer* (RA), illustrating the transition of conventional interventions toward precise immunological and host-directed strategies. RA vaccines comprised conventional inactivated bacterins, live attenuated vaccines- generated through virulence screening, serial passage, or targeted genetic engineering, recombinant subunit vaccines targeting conserved protective antigens, and next generation-vaccine platforms incorporating reverse vaccinology and advanced mucosal delivery systems. Emerging non-antibiotic intervention include hyperimmune egg yolk IgY for passive immunization, host-defense peptides with direct antimicrobial and immunomodulatory activities, phytochemicals and herbal therapeutics with antimicrobial and immunomodulation properties, microbiome- and nutrition-based approaches that enhance intestinal repair and immune homeostasis, and precision antimicrobial technologies including bacteriophage and other targeted platforms. Arrows within each panel indicate the general proposed mechanisms to the corresponding outcome, while dashed lines separate distinct or alternative strategies. Solid arrows show the principal outcome. Ag, antigen; AMP, antimicrobial peptide; ROS, Reactive Oxygen Species. The illustration is created using Biorender (https://BioRender.com/dg4e7rc).

The collective evolution of RA vaccines reflects a broader shift from empirical pathogen control to mechanism-driven immunological intervention (**Figure 3**). Although substantial progress has been achieved with multivalent bacterins, rationally attenuated mutants, recombinant antigens and advanced delivery platforms, most candidates remain constrained by the fundamental challenge of serotype diversity, with protection frequently restricted to homologous or closely related strains. Emerging evidence further suggests that effective immunity against RA cannot be fully explained by serum antibody responses alone, but likely requires coordinated interactions between systemic humoral immunity, cell-mediated responses, and mucosal immune defenses at the primary sites of bacterial entry. Consequently, future vaccine development should prioritize the identification of conserved protective determinants with integrated pangenomics, immunoproteomics, and structural vaccinology, coupled with delivery systems capable of eliciting durable mucosal and systemic immunity. In this context, emerging technologies successfully applied to several gram negative bacteria such as *Pasteurella multocida*, *Acinetobacter baumannii*, *Klebsiella penumoniae*, *Pseudomonas aeruginosa Salmonella* spp., *Shigella* spp., and several avian viral pathogens (i.e., Duck Tembusu virus and Duck hepatitis A virus) using multi-epitope vaccines, engineered OMVs and generalized modules for membrane antigens (GMMAs), bioconjugates, nucleic acid vaccines, and probiotic-vectored, viral-vectored, or bacterial ghost delivery systems provide valuable frameworks for RA vaccine innovation to overcome the persistent challenge of serotype-restricted immunity (210–221).

## 6 Alternative control and prevention strategies for *Riemerella anatipestifer*

Despite the advances and availability of vaccines for RA, disease control remains challenging due to extensive serotype diversity, limited cross-protective immunity, and the increasing emergence of multidrug-resistant strains (22, 23, 65, 171). These constraints highlight that neither vaccination nor antimicrobial therapy alone provide a universally effective solution for RA management. Consequently, research efforts have increasingly shifted to complementary interventions that target multiple facets of disease pathogenesis, including direct bacterial killing, enhancement of host antimicrobial defenses, reduction of excessive inflammatory responses, and restoration of host microbial homeostasis. Among these approaches, naturally derived bioactive compounds, immunotherapeutics, probiotics, bacteriophages, and nutritional immunomodulators have attracted considerable attention due to their antimicrobial properties while simultaneously influencing host immune function (**Figure 3**). In contrast to conventional antimicrobial drugs that primarily target bacterial viability, many of these interventions operate through multifaceted mechanisms, including disruption of bacterial membranes, interference with virulence-associated pathways, attenuation of pathological inflammation, and stimulation of innate and adaptive immune responses (136, 222, 223). This dual host- and pathogen-directed mode of action is particularly promising for RA infection, where disease severity is driven not only by bacterial proliferation but also by dysregulated inflammatory responses and tissue damage.

### 6.1 Passive immunization

Passive immunization represents one of the most direct methods for providing immediate protection against RA infection. This approach provides pre-formed pathogen-specific antibodies capable of rapidly neutralizing invading bacteria and facilitating their clearance before severe systemic disease develops (224). The therapeutic administration of 10–30 mg anti-RA IgY within 1 h of infection achieved complete protection and prevented RA-associated tissue pathology, whereas efficacy declined rapidly with delayed treatment. Prophylactic IgY administration completely protected ducklings challenged within 1–4 days after treatment and maintained partial protection up to 10 days, but declined substantially by day 14. In contrast, an inactivated vaccination showed the opposite kinetic profile while providing little to no protection during the first 4 days after immunization, but progressively increasing efficacy thereafter. Additionally, IgY-treated birds were protected from the characteristics pathological lesions associated with RA infection (225). Collectively, these findings suggest that passive IgY immunization may serve as an effective emergency intervention during outbreaks and as a temporary protective bridge in young ducklings until durable vaccine-induced immunity becomes established.

### 6.2 Host defense peptides

Naturally occurring host defense molecules known as antimicrobial peptides (AMPs), which function as evolutionarily conserved components of innate immunity, is another promising avenue. Unlike conventional antibiotics, AMPs combine rapid membrane-disruptive bactericidal activity with broad immunomodulatory functions that enhance pathogen clearance, immune recruitment, and tissue repair (226). Experimental administration of 100 µg epinecidin-1 (grouper derived peptide), anti-lipopolysaccharide factor (shrimp derived), and hepcidin TH1–5 and TH2-3 (tilapia derived), significantly reduced mortality in ducks challenged with RA. Direct membrane-disruptive activity of the peptides were also seen causing ultrastructural membrane rupture and efflux of intracellular contents. Beyond bacterial killing, peptide treatment further reduced bacterial colonization in the liver under both prophylactic and therapeutic regimens and was associated with modulation of infection-responsive host pathways, including Mn superoxide dismutase (MnSOD)-linked oxidative stress response in the brain and lipoprotein lipase (LPL)-associated metabolic alteration in the liver, indicating that their protective effects extend beyond direct antimicrobial activity to encompass regulation of host physiological response during infection (222). Together, these findings position AMPs as promising host-pathogen-directed therapeutics that simultaneously suppress bacterial burden and increase host resilience during RA infection. The demonstrated susceptibility of RA to avian β-defensin 2 additionally supports the potential of host defense peptides as biologically relevant antimicrobial effectors against RA (227). Nevertheless, their applicability remains constrained by limited *in vivo* validation, and the recent discovery of SspA-mediated degradation of LL-37 indicates that not all AMPs may be equally effective against RA, emphasizing the importance of elucidating bacterial AMP-evasion mechanisms and developing protease-resistant peptide therapeutics (100).

### 6.3 Phytochemical and herbal therapeutics

Plant-derived byproducts and bioactive compounds have also emerged as multifunctional alternatives capable of simultaneously targeting bacterial survival, host immunopathology and antimicrobial resistance. Phytochemicals often exhibit broad-spectrum biological activities that include membrane disruption, inhibition of virulence-associated pathways, antioxidant effects, and modulation of immune signaling networks (79). Several phytochemicals appear to exert their primary benefits through the modulation of host inflammatory responses during RA infection. Oral administration of 3,3’-diindolylmethane (200 mg/kg/day) increased survival rates by 14% while suppressing the expression of IL-17A, IL-6 and IL-1β (138). Berberine, an isoquinolone alkaloid from stems and roots of *Berberis*, *Hydrastis canadensis* and *Coptidis rhizoma*, administered at 200 mg/kg/day, increased survival by 31% and reduced the bacterial burden in the spleens and livers of RA-infected ducks while downregulating IL-17A. IL-17F, IL-6 and IL-1β, and upregulating IL-10 (132). Although with promising efficacies it has only been evaluated under controlled experimental conditions, the economic cost, large-scale administration, and drug residues must be taken into consideration. Thus, this warrants further studies prior to its application in routine duck production. Similarly, Yinzhihuang treatment, a formula rich in baicalin commonly extracted from *Scutellaria baicalensis Georgi*, inhibited RA growth *in vitro*, and injection with 0.02 g/kg in ducks reduced RA infection-associated mortality to 20% while modulating inflammatory and oxidative stress pathways through increased SOD and reduced MDA and NO levels (228). These results affirm that modulation of immunopathology and oxidative stress is an important therapeutic strategy to limit damage during systemic RA infection.

Beyond host-directed effects, several natural products exhibit potent antibacterial activity against RA. A recent study using a multi-herb Chinese herbal medicine formulation (*Taraxacum mongolicum*, *Atractylodes macrocephala*, *Fraxinus chinensis*, *Citrus aurantium*, *Coptis chinensis*, *Phellodendron chinense*, *Saposhnikovia divaricata*, and *Glycyrrhiza uralensis*) demonstrated potent antibacterial activity (minimum inhibitory concentration/MIC and minimum bactericidal concentration/MBC: 0.97 mg/mL), direct killing by disrupting bacterial cell structure and integrity, reduced hepatic bacterial burden, increased survival rate by 30%, and suppressed the expression of STAT3, and pro-inflammatory cytokines IL-17A, IL-17F, IL-8, IL-22, IL-1β, and IFN-γ (136). More recently, sophoraflavone G (from *Sophora flavescens*) showed a low propensity for inducing drug resistance and exhibited rapid bactericidal activity by binding to membrane phosphatidylglycerol and causing membrane disruption and leakage of intracellular contents. Therapeutic administration at 5–10 mg/kg significantly reduced bacterial burdens in the liver, lungs, and kidney, and the high dose demonstrated additional efficacy in the brain (229). Complementing these bactericidal strategies, chlorogenic acid found abundantly in fruits, vegetables and spices, restored doxycycline susceptibility in *Tet*(*X*)-positive multidrug resistant (MDR) RA strains through the suppression of resistance-associated pathways, highlighting the potential of phytochemicals not only as direct antimicrobials but also as resistance-modifying adjuvants capable of extending the utility of existing antibiotic classes (230, 231).

### 6.4 Microbiome- and nutritional-based interventions

Host resilience-based strategies aim to reduce disease susceptibility by enhancing mucosal barrier function, microbiome stability, and immune competence rather than directly targeting the pathogen. Early screening of goose-derived *Lactobacillus* isolates identified *L. salivarius* and *L. plantarum* strains with strong antagonistic activity against RA, primarily through organic acid production, while maintaining tolerance to acidic and bile-rich conditions, supporting their potential as probiotic alternatives to restore microbial homeostasis in waterfowl (232). Extending this observation, oral administration of the cell-free supernatant of *Lactobacillus plantarum* ZG-7 significantly reduced mortality and eliminated diarrhea in RA-infected Muscovy ducks. Mechanistically, the cell-free supernatant exerted direct antibacterial activity by increasing membrane permeability and inducing leakage of intracellular contents from RA bacterial cells, while reinforcing intestinal barrier function in RA-infected ducks as reflected by improved villus architecture and reduced crypt depth, upregulated expression of tight junctions (OCLN, CLDN-1, ZO-1) and mucosal transport channels (AQP2, AQP3, AQP10, SGLT1, PepT1), increased colonic mucin and decreased apoptosis of intestinal epithelial cells (141). Nutritional immunomodulators have similarly shown promise in augmenting host defense mechanisms. Dietary nano-selenium supplementation (0.3 mg/kg feed) markedly increased the efficacy of inactivated RA vaccination, resulting in complete protection, elimination of bacterial shedding or recovery in organs, increased heterophil phagocytic activity, elevated IgM and IgG responses, and upregulated expression of IL-2, IL-10, and IFN-γ following RA challenge (233). Collectively, these findings highlight the importance of host resilience, and strengthening mucosal defenses, immune competence, and microbiome stability as a sustainable complementary strategy for reducing RA susceptibility and improving vaccine performance.

### 6.5 Novel antimicrobial platforms

Recent advances have moved beyond conventional antibiotics to precision antimicrobial platforms that exploit previously underutilized vulnerabilities, including membrane homeostasis and susceptibility to phage-mediated killing. The synthetic guanidine derivative isopropoxy benzene guanidine (IBG) exhibited potent antibacterial activity through the disruption of membrane phospholipids, and collapse of the transmembrane proton gradient and low propensity for developing resistance, and displayed synergistic activity in reducing bacterial burdens in RA-infected ducks when administered at 4 mg/kg with gentamicin (4 mg/kg) (223). Similarly, an oregano-N-acetyl cysteine nanocomposite encapsulated within a chitosan nanoparticle demonstrated potent bactericidal activity against MDR RA isolates at concentrations well below cytotoxicity thresholds, highlighting the potential of nanotechnology-assisted antimicrobial delivery (234). However, the absence of *in vivo* validation in this formulation currently limits the assessment of its translational potential for RA control.

Precision biological antimicrobials, particularly bacteriophages and phage-derived endolysins, have gained attention as antibiotic alternatives against several bacterial pathogens in the past years. However, its practical application against RA remains constrained due to RA serotype diversity and the narrow and strain-dependent host ranges of phages, and uncertain pharmacokinetics in ducks. Although several RA phages have been previously isolated as cited, the first virulent RA phage (RAP44) genome sequence with molecular description was only reported in 2012 (235). Subsequent studies, as cited by Zhang et al, reported synergistic effects of phages in combination with antibiotics (i.e. RAP44 with ciprofloxacin against Raf71 or RAP15 with ampicillin against Raf63) against RA compared to treatment alone (236). These findings provide preliminary evidence that phage-antibiotic combination may enhance treatment efficacy against RA, although their benefits appear strain- and combination-specific and require broader validation. A 50-kb genomic island highly homologous to RAP44 was also identified in the RA chromosome and retained integrase-mediated integration, excision, and circularization functions, indicating that RAP44-like elements can contribute to phage-host genomic exchange (237). Five temperate RAP44-like phages with variable lytic spectra were later identified and demonstrated that experimental lysogenization conferred superinfection resistance and altered bacterial phenotypes, highlighting the challenges associated with temperate phages and strain-dependent susceptibility (236). More recently, the lytic bacteriophage vB_RanS_gdf21 and its derived endolysin LysGDF21 demonstrated potent antimicrobial activity against RA through the inhibition of biofilm formation and disruption of mature biofilms (238).

Addressing the inherent limitations of phages and its application in RA will require large libraries of strictly lytic phages screened against diverse field RA isolates. Rational cocktails containing phages with complementary host ranges and different bacterial receptors could broaden strain coverage and reduce resistance development, while phage-antibiotic combinations and engineered endolysins may provide additional complementary approaches. To address potentially rapid phage clearance, duck-specific pharmacokinetic and pharmacodynamic studies are needed to optimize administration route, dose, timing, repeated dosing, and sustained-delivery formulations. Unlike broad-spectrum antibiotics, phage-based interventions offer highly specific pathogen targeting while influencing the composition and functionality of gut commensal microbiota, thereby providing a valuable option for managing MDR (239, 240). Nevertheless, these approaches remain largely conceptual and preliminary for RA and require further validation of their safety, resistance dynamics, scalability, and efficacy under *in vivo* and commercial conditions.

Taken together, these emerging interventions underscore that sustainable RA control requires strategies that go beyond conventional vaccination and antibiotic use. Rather than focusing exclusively on pathogen elimination, recent advances increasingly explore the use of complementary mechanisms to enhance host resilience, modulate inflammatory responses, restore antimicrobial susceptibility, manipulate microbiome-mediated defenses and target novel bacterial vulnerabilities such as membrane integrity and phage susceptibility. While many of these approaches are still at the experimental stage, their diversity highlights a growing shift to multifaceted disease management capable of addressing both MDR and the biological complexity of RA pathogenesis. Future efforts should prioritize mechanistic validation, optimization of delivery systems, and evaluation of combinatorial approaches in production settings to identify integrated control strategies that provide durable, scalable, and economically viable protection against RA.

## 7 Conclusion and future perspectives

In summary, the evidence synthesized in this review reveals RA as a highly adaptable pathogen whose persistence in poultry production systems is driven by the dynamic interplay between pathogen evolution, host immune responses and intervention efficacy. Advances in epidemiology, comparative genomics, and molecular pathobiology have demonstrated that RA is not merely a causative agent of septicemia and polyserositis, but a host-adapted pathogen capable of coordinating adhesion, immune evasion, nutrient acquisition, stress adaptation, systemic dissemination, and neuroinvasion through interconnected virulence networks. These pathogen adaptive traits are furthered by the extensive serotype diversity of RA and the increasing emergence of multidrug-resistant strains, enabling RA to persist under both immune and antimicrobial selection pressures.

Concurrently, growing insights into avian immunology have fundamentally expanded our understanding of host defense against RA. Effective host protection appears to depend on the coordinated interaction of mucosal barrier integrity, secretory IgA responses, innate immune activation, humoral immunity, cellular effector mechanisms, and tightly regulated inflammatory pathways. Emerging evidence suggests that resistance to infection may depend not only on the capacity to control bacterial replication but also on the host’s ability to maintain immune homeostasis and limit excessive inflammation, thereby limiting tissue injury and systemic pathology. These findings support a more integrated view of RA infection in which disease progression reflects the outcome of continuous host-pathogen interaction rather than the action of individual virulence factors or immune responses alone.

Importantly, advances in vaccinology and alternative intervention strategies have exposed a recurring challenge in the field: the difficulty of achieving broad, durable, and field-relevant protection against a biologically diverse and highly adaptable pathogen until now. Although conventional bacterins and antimicrobial therapy have reduced the disease burden for decades, their effectiveness is increasingly constrained by serotype diversity, limited cross-protective immunity, and MDR strains. Similarly, while recombinant vaccines, DNA vaccines, OMV formulations, nanoparticle-assisted deliver systems, probiotics, phytochemicals, host defense peptides, bacteriophages and immunomodulators have demonstrated considerable promise, many are still limited by an incomplete understanding of protective immune mechanisms, insufficient field validation, and inadequate integration with the broader biology of host-pathogen interactions. Viewed together, these observations suggest that successful RA control cannot rely solely on pathogen elimination but must instead address the interconnected processes of pathogen adaptation, immune evasion, host protective immunity, and immunopathology. This perspective represents a shift from traditional pathogen-centric approaches to integrated prevention strategies designed to simultaneously reduce pathogen fitness, enhance host resilience, and promote durable protective immunity.

Future progress in RA prevention will depend on defining the determinants of durable and cross-protective immunity against a genetically and antigenically diverse pathogen. The integration of advances in genomic epidemiology, systems immunology, and functional pathobiology, together with emerging approaches such as next-generation vaccinology, pan-genomic antigen discovery, structure-guided antigen design, nanotechnology-assisted delivery systems, bacteriophage therapy, and microbiome-based modulation offer unprecedented opportunities to target specific pathogen vulnerabilities while enhancing host resilience. Equally important will be robust validation of protective immune correlates and the transition of promising alternative control strategies from experimental proof-of-concept studies to large-scale field validation in integrated disease management programs.
