# Supplementary material for: Deciphering defensive mechanisms in Riemerella anatipestifer infection: pathogen adaptation, host immune response and emerging strategies
Source: Front Immunol. 2026 Sep 8;17:1929344. doi: 10.3389/fimmu.2026.1929344 (PMC13595192; doi:10.3389/fimmu.2026.1929344)
Supplement: Supplementary file 1 [file Table1.docx]

| **Supplementary Table 1.** List of conventional, recombinant, genetically engineered, and next generation vaccines against *Riemerella anatipestifer*. | | | | | | | | |
| --- | --- | --- | --- | --- | --- | --- | --- | --- |
| **Vaccine type** | **Immunogenicity** | **Advantages** | **Limitations** | **Representative vaccine studies** | **Composition** | **Vaccine information** | **Results** | **References** |
| **Inactivated vaccine** |  |  |  |  |  |  |  |  |
| ***monovalent bacterin*** | Humoral and cellular immunity against homologous serotype | Safe; stable and easy to produce | Require booster doses for lasting protection; limited cross-serotype coverage | Serotype 1 broth culture bacterin (local strain 1081) | Formalin-inactivated bacteria + AlumOH adjuvant | **Dose:** 5 x 10^9^ CFU, given SC | Significant protection in homologous challenge but no cross-protection; vaccination at 2 weeks (PI= 88 at 7dpi, PI= 95.6 at 14 DPI); vaccination at 1 + 5 weeks (PI=81.8-95.8) | Pathanasophon et al., 1996 |
|  |  |  |  | Serotype 1 (RAf63) or Serotype 2 (RAf153 strain) | Inactivated bacteria + adjuvant (Seppic Montanide ISA 206) | **Formulation**: 1 x 10^9^ CFU/mL emulsified with adjuvant (1:1 v/v); **Dose:** 0.5 mL/ bird, given SC | PI= 70-100 (homologous challenge), 20-25 (heterologous challenge) | Zhai et al., 2013 |
|  |  |  |  | Serotype 2 (ZZY7 strain) | Formalin-inactivated bacteria + white oil adjuvant + levamisole (LMS) | **Formulation:** 6 x 10^10^ CFU/mL emulsified with adjuvant (100:200 v/v) + LMS (4mg/mL); **Dose:** 0.5 mL/ bird, given SC | ↑ IFN-γ, IL-4, IL-10 at 37 post vaccination; ↑ IL-2 at 23- and 37-post vaccination; PI=100; sustained IgG until 44 days post-vaccination | Zhang et al., 2014 |
|  |  |  |  | Serotype 1 bacterin (XT5 strain) | Formalin-inactivated bacteria + propolis adjuvant/oil emulsion adjuvant/AlumOH adjuvant | **Formulation:**  5 x 10^10^ CFU/mL emulsified with adjuvant (1:3 v/v); **Dose:** 0.5 mL/ bird, given SC | PI= 83-92 | Liang et al., 2024 |
| ***multivalent bacterin*** | Protection across multiple circulating serotypes | Offers broad protection; widely adopted in endemic areas | Reduced cross-protection when serotypes do not match | Serotype 1 (CH3) + serotype 2 (NJ3) + serotype 10 (HXb2) | Formalin-inactivated bacteria (1 volume of each strain) + adjuvant (Montanide ISA 70 VG) | **Formulation:** 10^9^ (CH3) + 5 X 10^8^ NJ3 + 5 x10^8^ HXb2 CFU/ bird**,** given SC as single immunization or prime-booster regimen | sustained antibodies until day 70; PI= 100 (serotypes 1 , 2, 10); **single immunization:** ↑ IFN-γ, IL-2, MHC-I, CD40, CD154 ; **prime-booster:** ↑ IL-4, IL-10, MHC-I, and MHC-II | Liu et al., 2013 |
|  |  |  |  | Serotype 1 + serotype 2 | Formalin-inactivated bacteria + paraffin oil/ Montanide ISA 70/ *Salmonella typhimurium* LPS | **Formulation:** equal volume of serotype 1 + serotype 2: 1 volume of oil/LPS**; Dose:** 0.5 mL/ bird, given SC administered at 2- + 6- weeks old | vaccine + LPS: ↑ NO_2_ and antibody titer, PI=100 (serotype 1 and 2), vaccine + paraffin oil: PI= 85 (serotype 1) and 90 (serotype 2); vaccine + montanide: ↑ antibody titer, PI=95 (serotype 1 and 2) | Elbehary et al., 2024 |
|  |  |  |  | Serotype 1 (XT5 strain) + Serotype 2 (WZX strain) | Formalin-inactivated + propolis adjuvant/oil emulsion adjuvant/AlumOH adjuvant | **Formulation:**  5 x 10^10^ CFU/mL emulsified with adjuvant (1:3 v/v); **Dose:** 0.5 mL/ bird, given SC | ↑ RA-specific IgY, IFN-γ, IL-2, IL-4, PI=90, alleviate pathological tissue damage, propolis or oil emulsion adjuvant ↑ protective efficacy | Liang et al., 2024 |
| ***Combined with other pathogens*** | Simultaneous protection against multiple diseases common in birds | Simplifies vaccination management; confers multi-pathogen immunity | May exhibit antigenic interference; protection can be reduced if vaccine serotypes do not match circulating strains | Serotype 1 + serotype 2 + serotype 5 + *E. coli* 078 | Formalin-inactivated bacteria | **Formulation:** 10^8^ CFU *E. coli* + 10^9^ CFU of serotype 1, 2, and 5**; Dose:** 0.5 mL/bird, given SC as prime-booster vaccination at 2- and 3-weeks | PI= 70-100 (4 weeks-old challenge), 100 (6 weeks-old challenge), 80-100 (8 weeks-old challenge) against challenge cultures of Serotype 1, 2, 5, and *E. coli* | Stoute et al., 2016 |
|  |  |  |  | Serotype 1 + serotype 2 + *Pasteurella multocida* serotype A5 + *P. multocida* serotype D2 | Formalin-inactivated bacteria + adjuvant (Montanide ISA 70 VG) | **Formulation:** 1 volume of each inactivated strain (adjusted to 1.4 x 10^10^ CFU/mL for RA serotypes, and 3.25 x 10^10^ CFU for *P. multocida* serotypes) + 7 volume of adjuvant; **Dose:** 0.5 mL/bird, given SC at 2-3 weeks of age (1st dose) and 4-6 weeks of age (2nd dose) | PI: 100 (both RA and *P. multocida* serotypes); sustained Ab titer for both pathogen until 12th weeks PPV | El-Rawy et al., 2020 |
|  |  |  |  | Serotype 1 + serotype 2 + Avian influenza H5N1 | Formalin-inactivated bacteria/virus + adjuvant (Montanide ISA 71 VG) | **Formulation:** equal volume of serotype 1 and serotype 2 (adjusted to 1.4 x 10^10^ CFU/ml each) + equal volume of inactivated H5N1 (10^8^ EID_50_^)^ in adjuvant (30/70 w/w)**; Dose:** 0.5 mL/ bird, given SC administered as single dose or prime-booster | PI= 75 (serotype 1 and 2) 3 weeks PPV, PI=85 (serotype 1 and 2) 3 weeks post booster vaccination; RA: peak Ab titer at 6-weeks (PPV), 5-weeks PB (booster at 3-weeks PPV), and 3-5 weeks PBV (booster at 8 week); AI: peak Ab titer at 5-weeks (PPV), 5-weeks PB (booster at 3-weeks PPV and booster at 8-weeks PPV) | Gamal et al., 2021 |
|  |  |  |  | Serotype 1 + serotype 2 + Duck hepatitis virus | Formalin-inactivated bacteria/virus + adjuvant (Montanide ISA 70 VG) | **Formulation:** 1.4 x 10^10^ CFU equivalents + ≥10^8^ EID_50_ virus in adjuvant (30:70, w/w); Dose: 0.5 mL/bird, given SC at day-old | PI= 72.7 (serotype 1 and 2); peak Ab at 6-weeks PV (RA) and 5-6 weeks (duck hepatitis virus) | Ali Abodalal et al., 2023 |
| ***inactivated genetically engineered mutants*** | High Ab titers and stimulate both humoral and cellular response | Broad cross protection, biologically safe | Require adjuvant, does not directly stimulate strong local mucosal immunity at the natural portals of entry | Random Tn4351 insertion mutants of RA CH3 (∆M946_1556, RA CH3∆M946_1603 or RA-M1, RA CH3∆M949_RS01035 or RA1062, RA CH3 ∆M949_RS01915 or RA1067) with defective LPS/O-antigen biosynthesis | Formalin inactivated mutant bacteria + adjuvant (Montanide ISA 70 VG); Inactivated mutant bacteria (RA1067 mutant) | **Formulation:** 10^9^ CFU/ duck, given SC once or prime-boost regimen; 5 x 10^8^ CFU in 0.3 mL/duck given SC at 7- and 21- days (RA1067 mutant) | PI= 87.5-100 (homologous serotype, WJ4 strain), 75-100 (RA serotype 2 Yb2 strain), and 87.5-100 (RA serotype 10 HXb2 strain) | Zou et al., 2015b, 2015a; Dou et al., 2017, 2018 |
| **Live attenuated vaccine** |  |  |  |  |  |  |  |  |
| ***virulence-screened strain*** | high Ab response, strong CMI, immune memory, native antigen presentation | rapid candidate selection, preserved field antigenicity, mass vaccination feasible, no extensive passaging required | Residual virulence risk, undefined attenuation basis, genetic instability, shedding and safety concern, limited cross-serotype protection | Serotype 1 (D15-RDA-92) or serotype 2 (D14-RDA-92); Serotype 1 + serotype 2 combination | Vaccine isolates were reconstituted in DW + 0.756g skim milk | **Formulation:** single strain: 10^8^ CFU/bird, given orally at day-old + 7 days-old; combined: 10^8^ CFU (serotype 1) + 10^7^ CFU (serotype 2)/bird, given orally at day-old + 7 days-old | Single strain: Homologous challenge PI= 60 (serotype 1) and 100 (serotype 2); combined: Serotype 1 challenge PI=70, serotype 2 challenge PI=100; ↑ sIgA in the trachea and duodenum of challenged birds at 28-DPI | Kang et al., 2018 |
| ***serially passaged attenuation*** | high Ab response, strong CMI, durable immunity, immune memory | Mimics natural infection, broad and long-term protection, mass vaccination feasible | Residual virulence, reversion risk, genetic instability, safety concerns, time-consuming development platform; limited cross-serotype protection | Serotype 2 (K-MLF 62-54) serially passage | Wild-type strain (62 serially passages in liquid and solid media with UV irradiation during the final 6 passages) | **Formulation:** 1:100 dilution of overnight culture in PBS; **Dose:** 0.5 mL/bird, given IM at 28 and 98 days | Slightly weaker CMI response vs. bacterin, highly persistent and long-lasting CMI (LTT response), similar serum Ab production with bacterin | Higgins et al., 2000 |
| ***live genetically engineered mutants*** |  |  |  |  |  |  |  |  |
| *Metabolic, regulatory and transport mutants* | Induce robust humoral immunity, including high titers of specific antibodies; Stimulates cellular immune responses, including T-cell mediated immunity | Highly attenuated and safe compared with wild-type strains; Strong and long-lasting immunity, often with a single dose | Residual risk of reversion to virulence must be monitored; Serotype coverage may be limited; cross-protection is variable | ∆B739_2187 mutant also named K10 strain (fis family transcriptional regulators of multiple antibiotic resistance promoter) | Allelic exhanged-derived RA CH-1 (Serotype 1) ∆B739_2187 mutant | **Dose:** 5 X 10^8^ CFU/bird, given IM | ↓ bacterial load in liver and brain, ↑serum IgG Ab at 14- and 21- PV; PI=100 (homologous challenge) | Zhao et al., 2016 |
|  |  |  |  | ∆*pncA* mutant (nicotinamidase) | Tn4351 transposon-derived RA Yb2 (Serotype 2) pncA mutant | **Dose:** 2.5 x 10^8^ CFU/bird, either once or prime-boost schedule given 7-days apart | PI= 80 (single immunization) and 90 (prime-boost immunization) in homologous challenge | Wang et al., 2016 |
|  |  |  |  | ∆B739_1343 mutant (TonB-dependent receptor/Iron acquisition) | Allelic exhanged-derived RA CH-1 ∆B739_1343 mutant | **Dose:** 108 CFU/bird, given IM | ↓ bacterial load in blood, liver and brain, ↑serum IgG Ab compared to inactivated RA-CH-1 vaccine at 7- and 14-days PV; PI=83.33 (homologous challenge) | Liu et al., 2018 |
|  |  |  |  | ∆Cas9 mutant (CRISPR-associated protein 9) | Conjugation-mediated RA YM (Serotype 1) Cas9 deletion mutant | **Dose:** 6 x 10^4^ CFU or 3 x 10^5^ CFU/bird, given either SC or IN | **SC immunization:** PI= 20 (6 x 10^4^ CFU dose) and 30 (3 x 10^5^ CFU dose) homologous challenge; **IN immunization:** PI= 0 (6 x 10^4^ CFU dose) and 80 (3 x 10^5^ CFU dose) homologous challenge | Wang et al., 2019 |
|  |  |  |  | ∆*fur* mutant (ferric uptake regulator, global iron/virulence regulator) | natural transformation-derived RA CH-1 *fur* knockout mutant | **Dose:** 10^9^ CFU/bird, given IM | no CS or significant histological lesions and bacteria is cleared within 3-days, ↑serum Ab IgY titer; PI = 89 (homologous challenge), no significant cross-protection (PI= 0-11.11, heterologous serotypes) | Huang et al., 2023 |
|  |  |  |  | ∆*phoP* mutant (Two-component system regulator) | Homologous recombination-derived RA YM phoP knockout mutant | **Dose:** 10^6^ and 10^7^ CFU/bird, given SC | PI=100 (homologous challenge), ↑serum Ab IgY titer at 7- and 14-days PV, ↑IL-2, IFN-γ, IL-4, IL-10 | Li et al., 2023 |
|  |  |  |  | ∆*ybiT* mutant (ATP-binding cassette transporter) | Natural transformation derived RACL-1 *ybiT* knockout mutant | **Dose:** 2 x 10^4-7^ CFU/ml in PBS, given 0.5ml IM | PI= 100 (homologous challenge) | Li et al., 2026a |
| *structural mutant* | Induce strong humoral immune responses (specific IgY antibodies); Stimulate cellular immunity, including T-cell responses | Highly attenuated; Robust and long-lasting immunity, often with a single administration | Residual risk of reversion to virulence; Safety must be carefully evaluated in immunocompromised or very young ducklings | LPS or bacterial antigenicity defective/inactivated gene mutants | Random Tn4351 insertion mutant RA∆604 (Serotype 1 CH-3 strain, M949_1360 mutant) | **Dose:** 5 x 10^8^ CFU/ml in 0.5mL PBS given prime-booster (7- and 14-days) | PI= 87.5 (homologous serotype, strain WJ4) | Yu et al., 2016 |
| *Secretion system mutant* | Induce strong humoral immunity (high levels of specific IgY antibodies); Elicit cellular and mucosal immune responses, providing broader protection | Extremely attenuated; Strong and long-lasting immunity with a single dose | Residual risk of reversion to virulence; Serotype coverage may still be limited; cross-protection is not guaranteed for all circulating serotypes | ∆*sprT* (type IX secretion system/ T9SS) | Allelic exchanged-derived RA YM ∆*sprT* | **Dose:** single dose 4 x 10^5^, 2 x 10^6^, 1 x 10^7^ CFU | PI= 60-100 (homologous serotype, YM strain given at 2x LD_50_ and 10x LD_50_) | Guo et al., 2017 |
| *Double-gene deletion mutant* | Robust humoral and cellular immune response, high mucosal response with IN administration | Enhanced safety while preserving immunogenicity, high protective efficacy | Genetic stability, long term immune durability, cross-serotype breadth beyond tested strains | ∆cas9∆sprA mutant | Homologous recombination derived markerless RA YM ∆cas9∆sprA double deletion mutant | **Dose:** single dose of 1.68 x 10^7^ CFU/duck (IM), 2.08 x 10^7^ or 2.08 x 10^8^ CFU/duck (IN) | **IM vaccine:** ↑ serum IgY Ab, ↑IFN-γ and IL-6, ↑ sIgA 2 weeks PV (trachea and lungs), PI= 100 (homologous serotype, RA YM) and 70 (Homologous serotype, RA A strain); **IN vaccine:** ↑ serum IgY Ab, ↑IFN-γ and IL-6, ↑sIgA 2 weeks PV (nasal cavity, trachea, lungs and rectum), PI= 100 (homologous serotype, RA YM) and 80-90 (homologous serotype, RA A strain) | Hou et al., 2026 |
| **Recombinant subunit vaccine** |  |  |  |  |  |  |  |  |
| ***conserved structural antigens*** | Strong humoral and moderate cellular responses; serotype-specific but some cross-protection possible | Safe (no live pathogen), easy to produce, can target conserved antigens | May require multiple doses and adjuvants; less effective than live vaccines | OmpA (Outer membrane protein A) | Serotype 2 recombinant OmpA (rOmpA) + CpG ODN adjuvant | **Formulation:** 100 µg rOmpA + 100 µg CpG ODN; **Dose:** 0.2 mL/ bird, given IM | ↑IFN-γ, IFN-α, IL-6, IL-12 in PBMC (2 weeks PPV); sustained serum Ab until 9 months PPV, ↓ pathological lesion, cross-serotype reaction (serotypes 1, 6, strain 56a) of vaccinated duck sera | Chu et al., 2015; Phonvisay et al., 2019 |
|  |  |  |  |  | rOmpA fused with duck IgY Fc + *Schisandra chinensis* polysaccharide adjuvant | **Formulation:** 200 µg/mL rOmpA + 200 mg/mL adjuvant; **Dose:** 0.2 mL/duck given SC at day 1, 7, and 14 | ↑CD4^+^ and CD8^+^ T lymphocyte count in peripheral blood, ↑serum Ab titer, ↑IL-4 and IL-2, PI = 86.7 (Homologous serotype, SD 01 strain) | Yang et al., 2019 |
|  |  |  |  |  | rGST-OmpA (recombinant Glutathione sulfatransferase fusion proteins of OmpA) + Adjuvant (Seppic Montanied ISA 773) | **Formulation:** recombinant protein (1mg/mL) in equal volume of adjuvant; **Dose**: 0.5mL/duck given SC on 7- and 14- day | elicit specific Ab production, PI= 14.3 (Serotype 15 34/90 strain) | Huang et al., 2002 |
|  |  |  |  | chaperonin GroEL | rGroEL + Montannide ISA VG adjuvant | **Dose:** 250 µg rGroEL/duck given SC as prime-booster regimen | PI = 50 (Serotype 1 WJ4), 37.5 (Serotype 2 Th4 and Serotype 10 HXb11) | Han et al., 2012 |
|  |  |  |  | p45N (45 kDA potential surface protein) | rGST-P45N' (recombinant Glutathione sulfatransferase fusion proteins of OmpA) + Adjuvant (Seppic Montanied ISA 773) | **Formulation:** recombinant protein (1mg/mL) in equal volume of adjuvant; **Dose**: 0.5mL/duck given SC on 7- and 14- day | elicit specific Ab production, PI= 14.3 (Serotype 15 34/90 strain) | Huang et al., 2002 |
| ***Immunoproteomic-screened cross reactive antigens*** | Induce cross-reactive Ab response | Naturally immunodominant targets | Immunoreactivity does not always translate to protection, may require high antigen doses or multi-antigen formulations | Serotype 1 (RAf63 strain) or Serotype 2 (RAf153 strain) subunit | Recombinant proteins (rCR1, rCR4, rCR5) + adjuvant (Seppic Montanide ISA 206) | **Formulation:**  individual recombinant protein (1.2mg/mL) + equal volume of adjuvant, combined recombinant protein at equal volume; **Dose:** 0.5 mL/ bird, given SC | **RAf63 challenge:** PI= 0-50 (individual protein) and 62.5 (combined protein); **RAf153 challenge:** PI=10-60 (individual protein) and 50 (combined protein) | Zhai et al., 2013 |
| ***Targeted surface and secreted virulence effectors*** | Strong Ab-mediated protection, potent antigen-specific protection | Neutralize key virulence mechanisms | May not fully neutralize multifactorial virulence | PaR1 or AS87_RS06600 (Type IX secretion system/T9SS) | rPaR1 + adjuvant (Montanide ISA 70 V) | **Dose:** 100µg rPaR1-adjuvant, given IM | PI = 20 (Heterologous serotype, Serotype 1 WJ4), 40 (homologous serotype, serotype 2 YB2), 0 (Heterologous serotype, serotype 10 HXb2) | Wang et al., 2023a |
|  |  |  |  | PorV (T9SS component) | rPorV + Freud's adjuvant | **Dose:** 200µg of PorV + Freuds complete adjuvant (primer)/Freuds incomplete adjuvant (booster)/ duck; **Dose:** primer and booster regimen | PI = 40 (Serotype 1 RA-YM), 75 (Serotype 5, RA-AH) | Li et al., 2026b |
| ***Antigens expressed under host-associated stress*** | Elicit humoral and cellular responses; can provide cross-serotype protection due to conserved iron uptake proteins | Targets essential survival pathways; potential broad cross-protection; relatively safe | Expression may be challenging; immunogenicity may be lower without strong adjuvants; less tested in large-scale trials | Iron restricted environment (novel proteins Riean_1750 and Riean_1752) | Recombinant proteins Riean_1750 + Riean_1752 + Freud's adjuvant | **Formulation:** 500µg recombinant proteins + Freuds complete adjuvant (primer)/Freuds incomplete adjuvant; (booster); **Dose:** 500µg/duck, given SC as primer (7-days) and booster (14- and 21 days) regimen | PI= 100 (homologous serotype 1) | Yang et al., 2013 |
| **Next-generation vaccines** |  |  |  |  |  |  |  |  |
| ***Next-generation antigen discovery*** | Robust humoral immune response | Highly targeted and precise vaccine design, rapid discovery of vaccine targets | Requires extensive downstream validation (i.e. *in vitro* and *in vivo*) | *In silico* analysis of outer membrane protein YaeT (OMP85 protein family) | Recombinant YaeT protein + adjuvant (complete/incomplete Freud's adjuvant) | **Formulation:** 1:1 ratio of YaeT protein and adjuvant; **Dose:** 0.2mg/duck**,** given SC as primer (7- days in complete Freud's) and booster (14-days in incomplete Freud's) regimen | ↑ high affinity Ab that could activate complement and promote opsonophagocytosis, PI=80 (RA CH1, serotype 1) | Guan et al., 2023 |
| ***Nanotechnology delivery systems*** | Robust systemic immunity, targeted mucosal immunity | Highly safe and biocompatible, highly effective at delivery of therapeutic payloads | complex formulation, less tested in large-scale trials | Calcium phosphate (CAP) nanoparticle conjugated with RA outer membrane vesicle (OMV) | RA OMV + CAP nanoparticles | **Dose:** 3762 µg or 2 x PD_50_ of CAP-OMV nanoparticle/ duck, given IN as prime-booster regimen | ↑sIgA and IgY titer, PI=100, no gross and biochemical indication of toxicity | Deka et al., 2023 |
|  |  |  |  | Engineered *Salmonella­-*derived OMV displaying PorQ, YaeT, or YiaD | RA recombinant antigens (PorQ, YaeT, or YiaD) + Salmonella OMV | **Dose:** 100 µg of OMV with PorQ / YaeT / YiaD per duck given IM as Primer and booster (14-days after primer) | PI= 70 (OMVs-PorQ), 80 (OMV-Yaet and OMV-YiaD) against serotype 2 (RA-SD-1); robust humoral and cellular immune response, reduced pathological damage and bacterial load | Zhou et al., 2026 |
| ∆, gene deletion(knockout) mutant; Ab, antibody; AlumOH, Aluminum hydroxide; CMI, cell mediated immunity; CS, clinical signs; DPI, day post infection; IM, intramuscular; IN, intranasal; LPS, lipopolysaccharide; LTT, lymphocyte transformation test; MHC, major histocompatibility complex; PBMC, peripheral blood mononuclear cells, PBS, phosphate buffer saline; PBV, post-booster vaccination; PD, proportional distance; PI, Protective Index; PPV, post primary vaccination; PV, post-vaccination; RA, *Riemerella anatipestifer*; SC, subcutaneous route. | | | | | | | | |
|  |  |  |  |  |  |  |  |  |
